# Host‐derived population genomics data provides insights into bacterial and diatom composition of the killer whale skin

**DOI:** 10.1111/mec.14860

**Published:** 2018-10-24

**Authors:** Rebecca Hooper, Jaelle C. Brealey, Tom van der Valk, Antton Alberdi, John W. Durban, Holly Fearnbach, Kelly M. Robertson, Robin W. Baird, M. Bradley Hanson, Paul Wade, M. Thomas P. Gilbert, Phillip A. Morin, Jochen B. W. Wolf, Andrew D. Foote, Katerina Guschanski

**Affiliations:** ^1^ Animal Ecology Department of Ecology and Genetics Evolutionary Biology Centre Uppsala University Uppsala Sweden; ^2^ Centre for GeoGenetics Natural History Museum of Denmark University of Copenhagen Copenhagen K Denmark; ^3^ Marine Mammal and Turtle Division Southwest Fisheries Science Center National Marine Fisheries Service National Oceanic and Atmospheric Administration La Jolla California; ^4^ SR3, SeaLife Response, Rehabilitation, and Research Seattle Washington; ^5^ Cascadia Research Olympia Washington; ^6^ Northwest Fisheries Science Center National Marine Fisheries Service National Oceanic and Atmospheric Administration Seattle Washington; ^7^ National Marine Mammal Laboratory Alaska Fisheries Science Center National Marine Fisheries Service National Oceanic and Atmospheric Administration Seattle Washington; ^8^ NTNU University Museum Trondheim Norway; ^9^ Science of Life Laboratories and Department of Evolutionary Biology Evolutionary Biology Centre Uppsala University Uppsala Sweden; ^10^ Section of Evolutionary Biology Faculty of Biology LMU Munich Munich Germany; ^11^ Molecular Ecology and Fisheries Genetics Laboratory School of Biological Sciences Bangor University Bangor Gwynedd UK

**Keywords:** Cetacea, contamination, metagenomics, microbiota, *Orcinus orca*

## Abstract

Recent exploration into the interactions and relationship between hosts and their microbiota has revealed a connection between many aspects of the host's biology, health and associated micro‐organisms. Whereas amplicon sequencing has traditionally been used to characterize the microbiome, the increasing number of published population genomics data sets offers an underexploited opportunity to study microbial profiles from the host shotgun sequencing data. Here, we use sequence data originally generated from killer whale *Orcinus orca* skin biopsies for population genomics, to characterize the skin microbiome and investigate how host social and geographical factors influence the microbial community composition. Having identified 845 microbial taxa from 2.4 million reads that did not map to the killer whale reference genome, we found that both ecotypic and geographical factors influence community composition of killer whale skin microbiomes. Furthermore, we uncovered key taxa that drive the microbiome community composition and showed that they are embedded in unique networks, one of which is tentatively linked to diatom presence and poor skin condition. Community composition differed between Antarctic killer whales with and without diatom coverage, suggesting that the previously reported episodic migrations of Antarctic killer whales to warmer waters associated with skin turnover may control the effects of potentially pathogenic bacteria such as *Tenacibaculum dicentrarchi*. Our work demonstrates the feasibility of microbiome studies from host shotgun sequencing data and highlights the importance of metagenomics in understanding the relationship between host and microbial ecology.

## 1 INTRODUCTION

The skin microbiome is an ecosystem comprised of trillions of microbes sculpted by ecological and evolutionary forces acting on both the microbes and their host (Byrd, Belkaid, & Segre, 2018; McFall‐Ngai, Henderson, & Ruby, 2005). Recent explorations have revealed a tight connection between many aspects of the host’s biology and the associated microbial community (Reviewed by Alberdi, Aizpurua, Bohmann, Zepeda‐Mendoza, & Gilbert, 2016; Bordenstein & Theis, 2015; Byrd et al., 2018; Koskella, Hall, & Metcalf, 2017; McFall‐Ngai et al., 2005). Although numerous intrinsic and extrinsic factors that influence the skin microbiome composition have been identified, the relative importance of these factors often appears to differ even between closely related host taxa (Kueneman et al., 2014; McKenzie, Bowers, Fierer, Knight, & Lauber, 2012; Wolz et al., 2017). Intrinsically, the host's evolutionary history, age, sex and health appear significant (Apprill et al., 2014; Chng et al., 2016; Cho & Blaser, 2012; Leyden, McGiley, Mills, & Kligman, 1975; McKenzie et al., 2012; Phillips et al., 2012; Ying et al., 2015). Extrinsically, both environmental factors, where a subselection of environmental microbes colonizes host skin (Apprill et al., 2014; Walke et al., 2014; Wolz et al., 2017; Ying et al., 2015), and socioecological factors, such as a host's social group and the level of interaction with conspecifics (Kolodny et al., 2017; Lax et al., 2014; Song et al., 2013; Tung et al., 2015), can play important roles.

Most microbiome studies to date are based on 16S ribosomal RNA gene sequences, a highly conserved region of the bacterial and archaeal genome (Hamady & Knight, 2009). However, in addition to potential biases in PCR amplification, in which low reliability of quantitative estimations arises due to mismatches in primer binding sites, PCR stochasticity and different numbers of 16S gene copies in each bacterial species (Alberdi, Aizpurua, Gilbert, & Bohmann, 2017), analysis of the 16S region can limit functional and taxonomic classification (Quince, Walker, Simpson, Loman, & Segata, 2017). In contrast, shotgun metagenomics can facilitate both high‐resolution taxonomic and functional analyses (Koskella et al., 2017; Quince et al., 2017; Ranjan, Rani, Metwally, McGee, & Perkins, 2016). The advent of affordable high‐throughput sequencing has seen an ever‐increasing number of population genomics studies in a wide range of study systems (e.g., Der Sarkissian et al., 2015; Jones et al., 2012; Nater et al., 2017; Poelstra et al., 2014). This affords an unprecedented opportunity to exploit sequencing data to secondarily investigate the microbial communities associated with the sampled tissue of their host (Ames et al., 2015; Lassalle et al., 2018; Mangul et al., 2016; Salzberg et al., 2005; Zhang et al., 2015).

Here, we explore the relative importance of extrinsic factors on the epidermal skin microbiome of free‐ranging killer whales (*Orcinus orca*) using shotgun sequencing data derived from skin biopsy samples of five ecologically specialized populations or ecotypes (Foote et al., 2016). Given the widespread geographical range (Forney & Wade, 2006) and variation in ecological specialization of killer whales, even in sympatry (Durban, Fearnbach, Burrows, Ylitalo, & Pitman, 2017; Ford et al., 1998), this species provides a good study system for exploring the effects of both geographical location and ecotype (a proxy for both sociality and phylogenetic history) on the skin microbiome. However, the opportunistic use of such data is also fraught with potential pitfalls. We therefore describe in detail, measures taken to disentangle potential sources of contamination from the true skin microbiome, thus providing a useful roadmap for future host microbiome studies that exploit host‐derived shotgun sequencing data.

## 2 MATERIALS AND METHODS

### 2.1 Study system

Throughout the coastal waters of the North Pacific, two ecotypes of killer whales are found in sympatry: the mammal‐eating “transient” and fish‐eating “resident” ecotypes (Filatova et al., 2015; Ford et al., 1998; Matkin, Barrett‐Lennard, Yurk, Ellifrit, & Trites, 2007; Saulitis, Matkin, Barrett‐Lennard, Heise, & Ellis, 2000). Four decades of field studies have found that they are socially and genetically isolated (Barrett‐Lennard, 2000; Filatova et al., 2015; Foote & Morin, 2016; Ford, 2009; Hoelzel & Dover, 1991; Hoelzel et al., 2007; Morin et al., 2010; Parsons et al., 2013). Killer whales have also diversified into several ecotypes in the waters around the Antarctic continent, including a form commonly observed hunting seals in the pack‐ice of the Antarctic peninsula (*type B1*), a form that feeds on penguins in the coastal waters of the Antarctic peninsula (*type B2*) and a dwarf form thought to primarily feed on fish in the dense pack‐ice of the Ross Sea (*type C*) (Durban et al., 2017; Pitman & Durban, 2010, 2012; Pitman & Ensor, 2003; Pitman, Fearnbach, & Durban, 2018).

### 2.2 Sample collection and data generation

**Figure 1:**
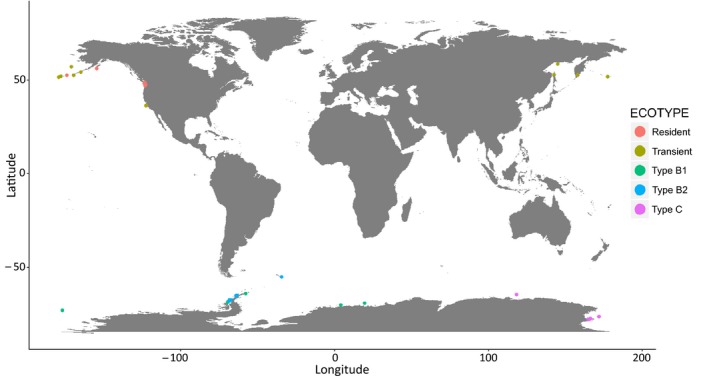
Map of sampling locations of the 49 samples of five killer whale ecotypes, from which skin microbiomes were included in this study. The Antarctic ecotypes primarily inhabit waters 8–16°C colder than the North Pacific ecotypes

We used the unmapped reads from a published population genomics study of killer whale ecotypes (European Nucleotide Archive, www.ebi.ac.uk/ena, Accession nos.: ERS554424–ERS554471; Foote et al., 2016), which produced low coverage genomes from a total of 49 wild killer whales, corresponding to five ecotypes: 10 samples each of the North Pacific fish‐eating *resident* and sympatric mammal‐eating *transient* ecotypes and 8, 11 and 10 samples, respectively, from Antarctic types *B1*,* B2* and *C* (see Figure 1 for the sampling locations). DNA was extracted from epidermal biopsies collected by firing a lightweight dart with a sterilized stainless steel cutting tip from a sterilized projector (e.g., Barrett‐Lennard, Smith, & Ellis, 1996; Palsbøll, Larsen, & Hansen, 1991) at the flank of the killer whale. As a study on captive killer whales found low variability in the taxonomic composition of the skin microbiome from different body sites (Chiarello, Villéger, Bouvier, Auguet, & Bouvier, 2017), small variation in the exact location on the flank from which the biopsy was taken should not bias our results. Biopsies were stored in sterile tubes at −20°C. At no point were biopsy samples in direct contact with human skin.

DNA extraction, library building and sequencing have been previously described (Foote et al., 2016). All laboratory work was conducted in a sterile flow hood to prevent contamination. Sequencing was performed at the Danish National High‐Throughput DNA Sequencing Centre within the University of Copenhagen. The facility is specifically geared for low‐quantity DNA library sequencing from ancient and environmental DNA. Samples of the same ecotype were pooled and sequenced across multiple sequencing lanes. Samples of different ecotypes were always run on different sequencing lanes, with the exception of several *type B1* and *B2* samples, which were initially grouped as “type B” (Pitman & Ensor, 2003), and some samples were therefore sequenced on shared lanes.

### 2.3 Sequencing read preprocessing

As a means to enrich the data set for bacterial sequences, we first used samtools v1.5 (Li et al., 2009) to remove all sequencing reads that mapped to the killer whale reference nuclear genome (Oorca1.1, GenBank: ANOL00000000.2; Foote et al., 2015) and mitochondrial genome (GU187176.1) with BWA‐mem (Li & Durbin, 2009). The remaining reads were adapter‐trimmed using adapterremoval V2.1.7 (Schubert, Lindgreen, & Orlando, 2016). We then removed duplicates generated during library indexing PCR by merging reads with identical sequences using in‐house python scripts (Dryad https://doi.org/10.5061/dryad.c8v3rv6). All reads with an average quality score <30 were filtered out using prinseq v0.20.4 (Schmieder & Edwards, 2011), and all reads of <35 bp were removed using adapterremoval.

### 2.4 Investigating contamination

Despite the precautions outlined above, contamination can be introduced at several stages of the sequence data generation and subsequently mistaken for the genuine host‐associated microbiome signal. Contaminating DNA can be present in laboratory reagents and extraction kits (Lusk, 2014; Salter et al., 2014). For example, silica in some commercial DNA spin columns is derived from diatom cells and therefore can be a potential source of contamination with diatom DNA (Naccache et al., 2013). However, the Qiagen QIAquick spin columns used in this study do not contain silica from biological material, according to the manufacturer. Cross‐contamination can also occur between samples processed in the same sequencing centre (Ballenghien, Faivre, & Galtier, 2017). The impact of contamination increases in samples with small amounts of true exogenous DNA and can swamp the signal from the host's microbiome (Lusk, 2014; Salter et al., 2014). Contamination can be assessed using negative controls (e.g., Davis, Proctor, Holmes, Relman, & Callahan, 2017). However, the data used in this study were initially produced with the sole focus on the host organism. Including extraction and library preparation blanks is not a routine procedure in population genomics studies based on high‐quality host tissue samples, and as such, blanks were not included in the laboratory workflow and hence not sequenced. Therefore, we instead implement an ad hoc workflow that attempts to differentiate between contaminant and real exogenous DNA from host species shotgun sequencing data.

#### 2.4.1 PhiX contamination

The contamination of microbial reference genomes by PhiX, which is used as a control in Illumina sequencing, is a known potential source of error in metagenomics studies using shotgun sequencing data (Mukherjee, Huntemann, Ivanova, Kyrpides, & Pati, 2015). Therefore, to avoid erroneous mapping of PhiX‐derived reads to contaminated genomes, we removed all reads mapping to the PhiX genome used by Illumina (NC_001422) with BWA‐mem 0.7.15 (Li, 2013) with default parameters.

#### 2.4.2 Environmental and laboratory contamination

If the amount of contamination (derived from laboratory reagents or environment) is relatively equal among samples, we expect the relative proportion of contaminant sequencing reads to be inversely correlated with the quantity of sample‐derived DNA; that is, low‐quantity DNA samples will be disproportionately affected by contaminant DNA sequences compared with high‐quantity samples (Lusk, 2014; Salter et al., 2014). We therefore estimated the correlation between the proportion of the total sequencing reads assigned to each microbial taxon (see below for how taxonomic assignment was conducted) and total DNA read count per sample (prior to removal of host DNA and before PCR duplicate removal). Microbial taxa for which the read count was significantly negatively correlated with the total number of reads per sample (including host DNA), that is those that consistently increased in abundance in low‐quantity DNA samples, were flagged as potential contaminants.

#### 2.4.3 Human contamination

To account for the possibility of contamination with human‐associated micro‐organisms, we next quantified the amounts of human DNA in our samples and used this as a proxy of human‐derived microbial contamination (see Supplementary Text, Supporting Information for the details of read processing). Only reads uniquely mapping to a single region of the genome with high quality (samtools ‐q 30 ‐F 4 ‐F 256) were retained, and we removed all duplicates using samtools rmdup in a single‐end mode. Human contamination levels were estimated by calculating the percentage of filtered reads mapped to the human genome (Supporting Information Table S1). We included these values as a covariate in statistical models as a way to, at least partially, control for contamination with human‐associated micro‐organisms.

#### 2.4.4 Known bacterial contaminants

Next, we investigated whether specific bacterial taxa that have previously been reported to be likely contaminants are present in our data set. Following read‐based analyses, we found that our samples were dominated by *Cutibacterium* (*Propionibacterium*) *acnes*, which is abundant on human skin (Byrd et al., 2018) and a known contaminant of high‐throughput sequencing data (Lusk, 2014; Mollerup et al., 2016). We therefore investigated the distribution of sequence identity between our *C. acnes* reads and the *C. acnes* reference genomes, with the expectation that human or laboratory contaminants would show high (close to 100) percentage identity, whereas killer whale‐derived *C. acnes* would be more divergent.

Additionally, we analysed data from a North Pacific killer whale sequenced at ~20× coverage in a published study, in which sample collection, DNA extraction and sequencing were entirely independent of our data production (Accession no: SRP035610; Moura et al., 2014). If *C. acnes* was present in these data, it would suggest that either it was a real component of the killer whale skin microbiome, or it was independently introduced as contamination in both studies.

Contaminant taxa are unlikely to be introduced in isolation. *Cutibacterium acnes* was confirmed to be a likely contaminant (see below), and we therefore removed all taxa with which it significantly co‐occurred. Using netassoc 0.6.3 (Morueta‐Holme et al., 2016), we calculated co‐occurrence scores between all taxon pairs in the raw taxa data set. We set the number of null replicates to 999 and corrected p‐values for multiple comparisons using the FDR method. From the resulting matrix, we selected taxa with the top 10% absolute significant co‐occurrence score with candidate contaminant taxa and removed these taxa from downstream analyses, along with *C. acnes*.

#### 2.4.5 Investigating sources of contamination

Finally, to ascertain the authenticity of our data and to estimate the level and possible source of contamination, we used sourcetracker v2.0.1 (Knights et al., 2011), a tool that implements a Bayesian classification model to predict the proportion of taxa derived from different potential source environments. This approach allowed us to compare the composition of the free‐ranging killer whale skin microbiome to other marine mammal skin microbiota and to a number of potential contaminating and environmental sources. We obtained data from public repositories and included microbial communities reflecting the marine environment (ocean water from Southern Ocean and the North Pacific, Sunagawa et al., 2015), other marine mammal skin (captive bottlenose dolphins *Tursiops truncatus* and killer whales along with the respective pool water samples and free‐ranging humpback whales, Bierlich et al., 2018; Chiarello et al., 2017), likely contaminants such as human skin and gut (Lloyd‐Price et al., 2017; Meisel et al., 2016; Oh et al., 2014) and laboratory contamination from commonly used reagents (sterile water, Salter et al., 2014) (Supporting Information Table S2). We attempted to specifically select sources that were obtained with the shotgun sequencing approach to avoid potential locus‐specific effects that can produce distinct microbiome profiles in amplicon‐based studies. However, only 16S rRNA amplicon data were available for the marine mammal skin and the laboratory contaminants, each study targeting a different region within this locus (Supporting Information Table S2). Therefore, to control for locus‐specific effects, we also included samples from a human skin 16S amplicon study (Meisel et al., 2016) and limited our data to reads mapping to the 16S rRNA gene for those comparisons (see Supporting Information for more detailed methodology of read processing).

We used the r package Vegan v2.4.6 (Oksanen, Guillaume Blanchet, Kindt, & Legendre, 2017) to calculate distances between microbiome profiles derived from these different data sets. After total sum scaling (TSS) normalization, abundance‐based Bray–Curtis and presence/absence‐based binary Jaccard distances were calculated and visualized using principal coordinate analysis. Subsequently, a subset of sources was used in sourcetracker and we used our killer whale data as sinks without applying rarefaction to either sink or source samples. We also repeated the sourcetracker analysis using free‐ranging humpback whales as the sink samples.

### 2.5 Taxonomic assignment

We used malt (MEGAN Alignment Tool) version 0.3.8 (Herbig et al., 2016) to create a reference database of bacterial genomes downloaded from the ncbi ftp server (ftp://ftp.ncbi.nlm.nih.gov/genomes/all/GCA, accessed 26 January 2017). We performed a semiglobal nucleotide–nucleotide alignment against the reference database. Semiglobal alignments are more suitable for assessing quality and authenticity criteria common to short‐read data and are also useful when aligning 16S rRNA data against a reference database such as silva (Herbig et al., 2016). Sequence identity threshold was set to 95% as per Vågene et al. (2018), but with a more conservative threshold of including only taxa with five or more aligned reads in subsequent analysis.

The nucleotide alignments produced in malt were further analysed in megan version 6.7.6 (Huson et al., 2016). Genomes with the presence of stacked reads in some genomic regions and/or large gaps without any mapped reads were flagged using a custom python script (Dryad https://doi.org/10.5061/dryad.c8v3rv6) and manually assessed in megan. This step was necessary to identify spurious and incorrectly supported bacterial taxa, which were removed from further analysis if they represented highly abundant species (Warinner et al., 2017). Taxonomic composition of the samples was interactively explored in megan, and the number of reads assigned to each taxon was exported for subsequent analysis.

Taxonomic assignment was also carried out using an assembly‐based approach. Filtered metagenomic sequences of all samples were merged to perform a co‐assembly using megahit 1.1.1 (Li, Liu, Luo, Sadakane, & Lam, 2015) with default settings and k‐list: 21,29,39,59,79. Assembly quality was assessed using quast 4.5 (Gurevich, Saveliev, Vyahhi, & Tesler, 2013). Contigs were subsequently mapped to reference bacterial genomes with mgmapper (Petersen et al., 2017) using best mode to assign taxonomy. The assembly file was indexed using BWA‐index and samtools‐faidx. BWA‐mem was subsequently used to map the reads of each sample back to the assembly contigs to finally retrieve the mapped reads using samtools‐view. Individual coverage values were calculated with bedtools 2.26.0 (Quinlan & Hall, 2010) and contig coverage table normalized using cumulative sum scale (CSS) as implemented in MetagenomeSeq (Paulson, Stine, Bravo, & Pop, 2013).

The sequencing data used in this study are rather shallow in terms of coverage of microbial taxa, corresponding to low coverage killer whale genomes (mean of 2×). Therefore, we explored how low sequencing depth may affect the inferred bacterial profiles. To this end, we used an independently sequenced 20× coverage *resident* killer whale genome (Moura et al., 2014). By drawing a random subset of reads from this genome using samtools, we compared the taxonomic composition of the microbiome of the same individual at 20x, 10x, 5× and 2× mean sequence coverage depth.

### 2.6 Diversity analyses

We calculated all diversity measures in Vegan (Oksanen et al., 2017), using reads that were assigned to the species level in megan. By focusing on taxa at the species level, we were able to explore the skin microbiome at a high resolution, an advantage of shotgun over amplicon‐based analyses. However, results of this analysis should be interpreted in the light of a species‐level focus, where we are exploring a small yet well‐resolved representation of the microbiome, which may potentially be enriched with pathogens and common environmental bacteria, rather than a holistic representation of the entire ecosystem.

To control for bias introduced by varying genome size (species with larger genomes show higher read counts, which are translated into higher abundance scores; Warinner et al., 2017), we divided all read counts by the size of the respective full bacterial genome. If the taxon was mapped to the level of the strain, we divided the read count by the published genome size of that strain; if identified to the species level, we divided the read count by the average genome size across all published strains of that species.

Beta diversity was explored using two dissimilarity matrices in Vegan: abundance‐based Bray–Curtis and presence/absence‐based binary Jaccard distances. To assess the strength and significance of ecotype and geographical location (longitude and latitude) in describing variation in community composition, we conducted permutational multivariate analysis using the function adonis in Vegan. We controlled for differing depths of coverage between samples using two techniques. First, we used genome size‐controlled data (see above) and included the number of reads mapping to the species level as a covariate. Second, TSS normalization of the genome size‐controlled data was conducted, followed by conversion to the Bray–Curtis distance matrix. TSS normalization is irrelevant for the presence/absence data, as only species presence, rather than species abundance, is retained in the binary presence/absence matrix. As a result, three models were explored: two Bray–Curtis models with differing depth control techniques and one Jaccard model using read counts as covariate. Each model consisted of the following covariates: latitude (numeric), longitude (numeric), ecotype (categorical) and percentage human contamination (numeric), with library size included only when TSS normalization was not used. For each model, residuals were permuted 9999 times. We used the function betadisper (Vegan), followed by analysis of variance (ANOVA) to test for homogeneity of group dispersions. betadisper can be used to ensure that (a) the adonis model results are not confounded by heterogeneous variances (Anderson, 2001) and (b) to make biological inferences about between‐group variance in community composition.

We used the function capscale from the Vegan package to perform principal coordinate analysis (PCoA). The four bacterial taxa that described the most variation on PCoA1 and the four that described the most variation on PCoA2 were designated as “driving taxa.” We therefore classified a total of eight unique driving taxa that describe individual differences in microbiome composition (Supporting Information Table S4).

### 2.7 Network analysis

To venture beyond single microbial taxa and explore microbial interactions that include interspecific dynamics, we expanded our analyses to networks of bacterial communities associated with the driving taxa identified through the PCoA. Using netassoc (Morueta‐Holme et al., 2016), we compared the observed partial correlation coefficients between taxa with a null distribution estimated from identical species richness and abundances as the observed data. Again, taxa co‐occurrence scores were calculated between all taxon pairs in the raw data set, with null replicates set to 999. The FDR method was used to correct *p*‐values for multiple comparisons. From the resulting matrix of significant co‐occurrence scores, we selected the 20 taxa with the highest absolute co‐occurrence score for each of the eight unique driving taxa. We created a new matrix including only these taxa and visualized co‐occurrence networks.

### 2.8 Functional profiling

Community composition can be a poor predictor of the functional traits of the microbiome, due to processes such as horizontal gene transfer (HGT) between bacterial taxa, which can decouple species composition and function (Koskella et al., 2017). Shifting focus from the taxonomic composition to the genic composition of the microbiome reduces the impact of HGT on functional characterization (Koskella et al., 2017).

To explore functional profiles of the samples, we used diamond v0.9.10 with default parameters (Buchfink, Xie, & Huson, 2015) to create a reference database of nonredundant protein sequences from fully sequenced bacterial genomes downloaded from the nbci ftp server (https://ftp.ncbi.nlm.nih.gov/genomes/genbank/ accessed 9 March 2017). Nucleotide‐to‐amino acid alignments of the sample reads to the reference database were performed in diamond and the top 10% of alignments per query reported. The megan tool daa‐meganizer was then used to assign reads to proteins based on the diamond alignments and to assign functional roles to these proteins using the seed (Overbeek et al., 2005) and eggnog (Huerta‐Cepas et al., 2017) databases. Since one protein can have more than one function, it is possible for one read to be assigned to multiple functional subsystems. The raw count data (number of reads assigned to functional subsystem) were exported from megan and further processed in r. To control for differences in library depth, read counts per functional group were normalized by total read numbers mapping to seed or eggnog terms. We used principal component analysis (PCA) performed in the r package prcomp to visualize differences in functional groups between individuals.

We additionally performed an assembly‐based functional profiling to overcome the individual weaknesses of both assembly‐ and read‐based methodologies (Quince et al., 2017). *Ab initio* gene prediction was performed over the metagenomic assembly using prodigal 2.6.3 (Hyatt et al., 2010). The list of predicted gene sequences was indexed using BWA, and samtools was used to map the reads of each sample back to the gene sequences. We used bedtools 2.26.0 (Quinlan & Hall, 2010) to calculate individual coverages. Gene coverage table was subsequently CSS normalized using metagenomeseq (Paulson et al., 2013).

### 2.9 Diatom association analyses

Antarctic killer whales are often observed to have a yellow hue, which has been attributed to diatom coverage (Berzin & Vladimirov, 1983; Pitman & Ensor, 2003), and identifiable individuals have been observed to transit from this yellow skin coloration to a “clean” skin condition (Durban & Pitman, 2012). This change is hypothesized to occur during brief migrations to subtropical latitudes, where turnover of the outer skin layer takes place with a reduced thermal cost (Durban & Pitman, 2012). If this hypothesis is correct, diatom abundance should be correlated with skin age and coloration (Durban & Pitman, 2012; Hart, 1935; Konishi et al., 2008). Interindividual variation in microbiome profiles within the Antarctic ecotypes could therefore reflect variation in the age of the outer skin layer. During network analysis, we identified a possible association between key bacterial taxa driving between‐sample differences in community composition (*Tenacibaculum dicentrarchi*) and bacterial taxa associated with diatoms. Following from our observations that three samples from Antarctic ecotypes had high abundances of *T. dicentrarchi* and that in the PCoA these samples were differentiated from most other samples, we investigated the link between observed diatom coverage, abundance of *T. dicentrarchi* and abundance of other algae‐associated bacterial taxa. We conducted qualitative colour grading of *type B1* and *type B2* individuals using photographs taken at the time of biopsy collection, ranging from “clean” through to “prominent” yellow coloration.

We used two methodologies to quantify the level of diatom DNA in our samples. First, we used malt and megan in the same taxonomic pipeline as previously described, but with a reference database comprised of ncbi RefSeq nucleotide sequences from the diatom phylum Bacillariophyta (downloaded 30 October 2017). To date, only seven diatom reference genomes are available; thus, identification at the species level was not attempted. Instead, numbers of reads mapping to Bacillariophyta were exported and further processed in r. Raw diatom read counts were converted to the proportion of the total number of sequencing reads per sample. Second, we aligned all reads against the silva rrna database (release 128, Quast et al., 2013) using BWA‐mem 0.7.17, retained reads mapping with >10 mapping quality with samtools and used uclust (Edgar, 2010) in qiime 1.9.1 (Caporaso et al., 2010) to assign taxonomy based on the silva 18S database at 97% similarity. From the resulting OTU table, we retained reads that matched to known diatom taxonomy.

We explored the correlation between latitude (by grouping North Pacific and Antarctic ecotypes) and proportion of reads per sample mapping to diatoms using a generalized linear model with a quasi‐Poisson error structure and log link. As covariates, we included the longitude, number of reads mapping to the bacterial species level to control for library size and number of human reads to control for human‐associated microbial contamination. Using the same model structure, we then tested the correlation between the proportion of reads per sample mapping to diatoms and the presence/abundance of *T. dicentrarchi* reads, as well as the correlation with the presence of known algae‐associated bacterial taxa (including *T. dicentrarchi, Cellulophaga baltica*,* Formosa* sp. *Hel1_33_131*,* Winogradskyella* sp., *Marinovum algicola*,* Agarivorans gilvus*,* Pseudoalteromonas atlantica* and *Shewanella baltica*: Bowman, 2000; Amin, Parker, & Armbrust, 2012; Goecke, Labes, Wiese, & Imhoff, 2013; Goecke, Thiel, Wiese, Labes, & Imhoff, 2013, incorporated as a binary variable).

## 3 RESULTS

Metagenomic profiles from the skin microbiome of 49 killer whales from five ecotypes (Figure 1) were successfully reconstructed using shotgun sequencing data from DNA extracted from skin biopsies. Of the reads retained following our stringent filtering procedure, but before our investigations into *Cutibacterium acnes* as a possible contaminant, 8.20% (*n* = 7,984,195) were assigned to microbial taxa using the read‐based approach, with 2.45% (*n* = 2,384,587) assigned at the species level (see Dryad repository https://doi.org/10.5061/dryad.c8v3rv6). Overall, 845 taxa of microbes were identified. The co‐assembly yielded a 33.01‐Mbp‐long metagenome comprised of 45,934 contigs (N50 = 970 bp, average = 730 bp, max = 48,182 bp). Taxonomy was assigned to 41.73% of the contigs. Results from the assembly‐based approach were concordant with the read‐based results, and we therefore report only the latter.

### 3.1 Investigating contamination

On average, 0.16% of reads (range 0.01%–5.43%) mapped to the human genome (Supporting Information Table S2), suggesting the presence of human contamination and making it possible that human‐derived bacteria were present in our data set. After correcting for multiple testing, we found no significant negative correlation between the proportion of reads assigned to each bacterial taxon and the total number of sequenced reads (Supporting Information Figure S1). Negative trends (although not significant) between some bacterial taxa and the total number of sequenced reads were largely driven by one outlier sample with the lowest coverage (B1_124047). Following the deduplication step of our processing pipeline, these taxa were no longer present in the data set, as they fell below our defined threshold of five aligned reads in MALT (Supporting Information Figure S2).

*Cutibacterium acnes* was identified as the most abundant bacterial taxon, with an average abundance of 39.57% (*SD *= 24.65; Supporting Information Figure S3), but it may have been introduced via human or laboratory contamination (Lusk, 2014). Percentage identity to the human‐derived *C*. *acnes* genome was 100% for 245 and over 97% for 505 of the 527 contigs identified as *C*. *acnes* by MGMapper (Supporting Information Figure S4), supporting the idea of a likely exogenous source of *C. acnes*. Killer whale samples pooled by ecotype were sequenced across multiple sequencing lanes, allowing us to investigate whether contamination with *C*. *acnes* was introduced at the sequencing step. Relative *C*. *acnes* abundance per sample was highly similar between sequencing lanes (coefficient of variation = 0.076; Supporting Information Figure S5), suggesting that the contamination occurred prior to sequencing. However, *C*. *acnes* was also present to a high abundance (18.06% of reads aligning at species level) in the independently sequenced *resident* killer whale (Moura et al., 2014), suggesting that contamination with *C. acnes* was not specific to our workflow. We concluded that there was a high probability that *C acnes* was a laboratory contaminant and therefore removed all *C. acnes* reads/contigs from our data set before continuing with analysis.

#### 3.1.1 Network analysis results for *C. acnes*‐associated taxa

Following its identification as a likely contaminant, we used network analysis to identify and remove the top 10% of species which significantly co‐occurred with *C. acnes,* which corresponded to co‐occurrence scores above the absolute value of 1,000 (Supporting Information Figure S6). Overall, 82 species were removed (Dryad https://doi.org/10.5061/dryad.c8v3rv6), many of which are known human‐associated bacterial taxa. Following this filtering step, one *type C* sample had no remaining taxa. We therefore excluded this sample from further analyses.

#### 3.1.2 Metagenomic affinities of wild killer whale skin microbiome

**Figure 2:**
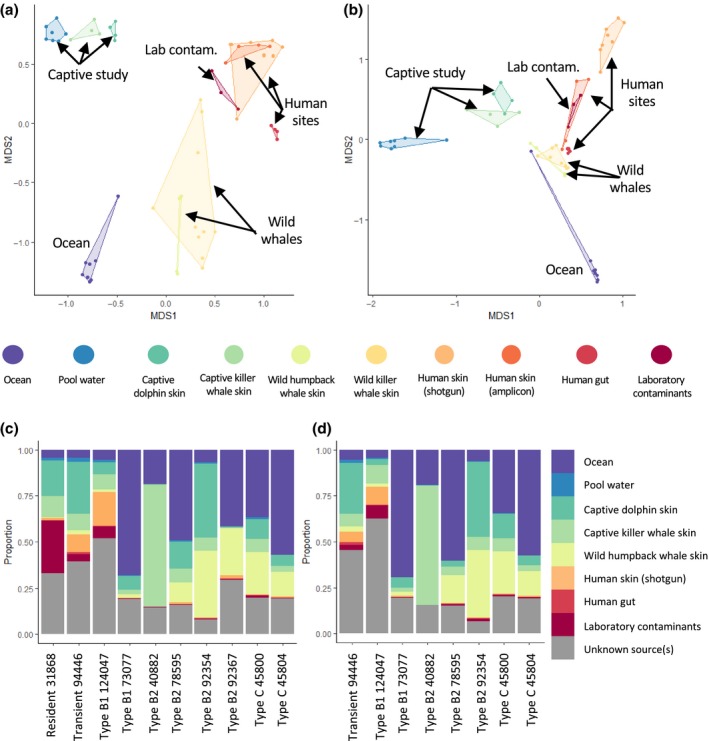
Composition of the wild killer whale skin microbiomes and other published microbiomes, for samples with ≥50 taxonomy assigned 16S reads. Principal coordinate analysis of Jaccard binary presence/absence distances before (a) and after (b) filtering of *C. acnes*‐associated taxa from the wild killer whale data. Proportions of sources contributing to each killer whale sample, represented by columns, from SourceTracker analysis before (c) and after (d) filtering of *C. acnes‐*associated taxa. * in (c) denotes samples that were excluded after *C. acnes* filtering due to low read numbers

Only 10 killer whale samples had 50 or more 16S reads with assigned SILVA taxonomy (eight killer whale samples remained after filtering for *C. acnes‐*associated taxa, Figure 2). Overall, prior to *C. acnes* filtering, the killer whale data set had 273 taxa in common with the data set of 2,279 bacterial taxa derived from sources (e.g., human, marine mammal and environmental samples, see Section 2.4.5). After filtering for *C. acnes* and associated taxa, 236 of the 273 killer whale‐associated taxa remained. Free‐ranging killer whale and humpback whale skin microbiomes overlapped on the principal coordinates, independent of the applied distance measure and the presence of *C. acnes*‐associated bacteria (Figure 2a, Supporting Information Figure S7a,b). In contrast, data from the captive study, including killer whale and captive dolphin skin samples and their pool water, clustered separately from all other studies. General separation by sequencing approach (i.e., shotgun versus amplicon) was not observed: for instance, amplicon‐ and shotgun‐sequenced human samples grouped together (Figure 2a,b). It is therefore possible that the separation of the captive study samples is due to either the use of a specific 16S target locus or other factors associated with captive versus wild environments (note that the pool was filled with sea water from the Mediterranean Sea; Chiarello et al., 2017).

The three marine mammal species formed one cluster irrespective of the study on the third dimension in the abundance‐based Bray–Curtis distance analysis (Supporting Information Figure S7c,d), suggesting that there is a common factor to the marine mammal skin microbiome composition. Importantly, the free‐ranging killer whale microbiome profiles generally grouped away from the human skin samples, gut samples and laboratory contaminants. They were also separated from the ocean water samples, suggesting that the killer whale skin microbiomes characterized in our study represent a microbial community that is clearly distinct from surrounding ocean water. Here, it is noteworthy that filtering of our data for *C. acnes*‐associated taxa at the genus level is highly conservative and also removes a number of microbial taxa that are abundant in the marine environment, as they belong to the same genera as some *C.  acnes*‐associated species. Samples representing laboratory contamination consistently clustered with the human skin samples (Figure 2a,b, Supporting Information Figure S7), suggesting that one source of contaminants in laboratory work are human‐associated skin microbes. All results presented above were confirmed with a larger data set that included 16 killer whale samples with at least 20 bacterial 16S reads with SILVA taxonomy assignment (Supporting Information Figure S8).

Based on the principal coordinate analysis and for greater clarity of presentation, we restricted the selection of samples that were used as sources in the SourceTracker analysis to captive dolphin skin (*n* = 4), captive killer whale skin (*n* = 4), water from the captive killer whale pool (*n* = 4), wild humpback whale skin (*n* = 4), Southern Ocean water (*n* = 4), human gut (*n* = 4), shotgun‐derived human skin data from a sebaceous site (*n* = 4) and laboratory contamination (*n* = 3; the fourth sample had <20 16S reads and was excluded from the analysis) (Supporting Information Table S2). The SourceTracker results supported those of the principal coordinate analysis (Figure 2c,d), with human skin taxa contributing on average only 3.4% to the wild killer whale skin microbiome (range 0.0%–18.4%). This percentage decreased to 2.2% (range 0.0%–9.6%) after filtering out *C. acnes*‐associated taxa. The contribution of laboratory contaminants was also low (average 4.2%, range 0.0–28.6) in all but one resident killer whale individual (31868), which was removed after *C. acnes* filtering due to low (<50) read numbers (average 1.7%, range 0.0%–7.1% after removal of *C. acnes*‐associated taxa). The sources contributing the most to the free‐ranging killer whale skin microbiomes after removing *C. acnes*‐associated taxa included Southern Ocean (mean 32.3%, range 4.5%–69.4%), humpback whale skin (11.9%, range 0%–36.7% in), captive killer whale skin and captive dolphin skin (mean 13.2%, range 2.1%–64.8% and mean 12.5%, range 0.2%–40.8%, respectively). A high proportion of taxa observed in free‐ranging killer whales could not be assigned to any of the sources included in the analysis (“Unknown,” mean >25%). These taxa may represent uncharacterized diversity specific to the wild killer whale skin microbiome, a source that was not included in our analysis, for example ocean water collected at the same time as the killer whale skin biopsies or marine mammal skin taxa that are poorly characterized by the 16S locus targeted in other marine mammal microbiome studies.

To verify the SourceTracker results for free‐ranging killer whale samples studied here, we also ran SourceTracker using the four wild humpback whales as the sink samples while assigning free‐ranging killer whales as a source (Supporting Information Figure S9). Two humpback whales sampled early in the foraging season around the Antarctic Peninsula closely resembled the wild killer whale profiles, containing a mixture of taxa attributed to the wild killer whale skin (41.7% and 65.3%), the captive dolphin skin (31.1% and 2.7%) and unknown sources (21.3% and 24.5%). In contrast, the microbiome of the two humpback whales sampled late in the Antarctic foraging season was dominated by Southern Ocean taxa (both >95%). This is consistent with the temporal variation in the complete humpback whale data set reported by Bierlich et al., (2018). Overall, the detailed analyses of contributing sources of the killer whale skin microbiome revealed a large proportion of taxa that are also found on the skin of other marine mammals and an important contribution of environmental ocean water taxa. This is in line with previous reports that found a significant contribution of sea water to, yet distinct composition of, marine mammal microbiomes (Bik et al., 2016). Expected contaminating sources, such as human skin and laboratory contaminants, contributed only a small proportion to our killer whale skin microbiome data obtained from host shotgun sequencing.

### 3.2 Taxonomic exploration

**Figure 3:**
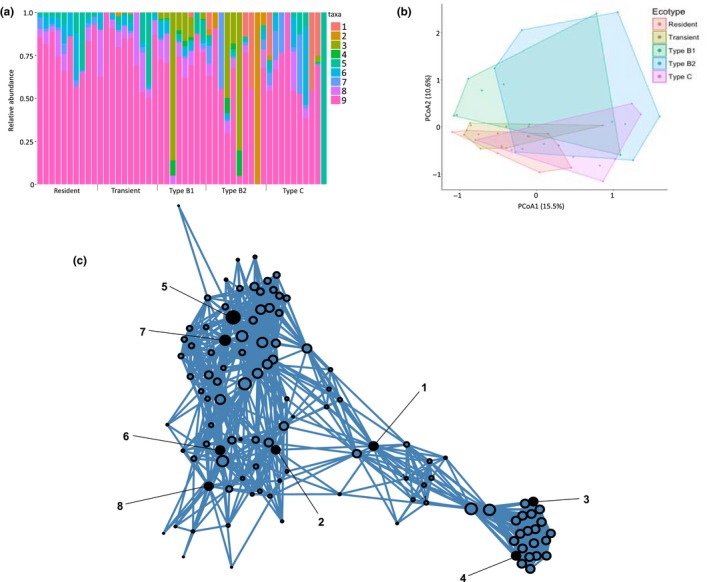
(a) Proportion of driving bacteria per individual, after data filtering. Individuals, represented by columns, are grouped by ecotype, and the relative proportions of bacterial taxa are indicated by column shading (1, *Tenacibaculum dicentrarchi*; 2, *Paraburkholderia fungorum*; 3, *Pseudoalteromonas haloplanktis*; 4, *Pseudoalteromonas translucida;* 5, *Acinetobacter johnsonii*; 6, *Pseudomonas stutzeri*; 7, *Stenotrophomonas maltophilia*; 8, *Kocuria palustris*;* and* 9, other). (b) Beta diversity between ecotypes illustrated as a Bray–Curtis PCoA estimated from read counts. (c) Positive co‐occurrence network built from a co‐occurrence matrix of all species, subsetted to the eight driving taxa (black nodes numbered as above) and their top 20 positive and significant co‐occurring species. Only species with a significant co‐occurrence score of >800 are shown

Read‐based and assembly‐based approaches produced concordant taxonomic profiles. The most abundant constituents of the killer whale skin microbiome at the phylum level were Proteobacteria, Actinobacteria, Bacteroidetes and Firmicutes (Supporting Information Figure S3a), which have been identified in previous studies of baleen whale skin microbiota (Apprill et al., 2014; Shotts, Albert, Wooley, & Brown, 1990), including through 16S amplification of skin swabs from captive killer whales under controlled conditions (Chiarello et al., 2017). At the species level, we found a high level of interindividual variation (Figure 3a, Supporting Information Figure S3b), as previously found for four captive killer whales housed in the same facility (Chiarello et al., 2017).

Subsetting an independently sequenced *resident* killer whale genome to lower sequencing depth, we inferred that while five most common taxa were found in similar proportions in high and low coverage data, the identification of rarer taxa became more stochastic at lower sequencing depths (Supporting Information Table S3). Our results may therefore suffer from this bias associated with low coverage data, which would be most prominent in the presence/absence‐based analyses. As a means to control for this bias, we include library size as a covariate in models investigating beta diversity.

### 3.3 Diversity analyses

Human contamination was not a significant driver in the models exploring beta diversity (Table 1), explaining at most 2% of the variation in taxonomic composition in each model. Ecotype was a significant variable in all models, explaining 10%–11% of variation in the data (Table 1). Latitude was significant in both Bray–Curtis models but not in the Jaccard presence–absence model. Where significant, it explained 4%–5% of variation in the data (Table 1). Longitude was not significant in any of the models. *Betadisper* analysis revealed no significant heterogeneity in the variation of community composition between ecotypes (non‐TSS normalized Bray–Curtis: d.f. = 4, *F* = 0.52, *p* = 0.72; TSS normalized Bray–Curtis: d.f. = 4, *F* = 1.74, *p* = 0.16; binary Jaccard: d.f. = 4, *F* = 0.63, *p* = 0.64). This suggests that between‐individual variation in microbial composition is similar among ecotypes.

**Table 1:** Factors influencing the killer whale skin microbiome

	(a) Bray–Curtis			(b) Bray–Curtis (TSS normalized)			(c) Binary Jaccard		
	*F*	*r* ^2^	*p*	*F*	*r* ^2^	*p*	*F*	*r* ^2^	*p*
Latitude	1.8	0.04	**0.01**	2.35	0.05	**<0.01**	1.4	0.03	0.05
Longitude	0.89	0.02	0.62	0.89	0.02	0.61	0.99	0.02	0.45
Ecotype	1.36	0.11	**<0.01**	1.35	0.11	**0.02**	1.23	0.10	**0.03**
Library size	1.33	0.03	**<0.01**	–	–	–	1.20	0.02	0.23
Human contamination	1.13	0.02	0.35	0.59	0.01	0.89	1.04	0.02	0.42
Residuals		0.79			0.81			0.80	
Total		1			1			1	

Note

Results of Adonis models using genome size‐controlled species data. (a) Bray–Curtis model with library size included as a covariate; (b) TSS normalized Bray–Curtis model; and (c) binary Jaccard model with library size included as a covariate. Significant factors are highlighted in bold.

The Bray–Curtis PCoA explained more variation than Jaccard (24.13% vs. 16.06% on the first two axes), and we therefore focus on the Bray–Curtis results. A network based on significant co‐occurrences between eight bacterial taxa driving variation at the individual level (Supporting Information Table S4) and the top 20 co‐occurring taxa for each of the driving taxa showed clearly differentiated and distinct community groups (Figure 3). Further investigation found that three of the taxa showing the highest co‐occurrence scores with the driving taxon *T. dicentrarchi* (*Formosa sp. Hel1_33_131, Cellulophaga algicola* and *Algibacter alginolytica*) are associated with algae (Becker, Scheffel, Polz, & Hehemann, 2017; Bowman, 2000; Sun et al., 2016).

### 3.4 *Tenacibaculum dicentrarchi* and diatoms

**Figure 4:**
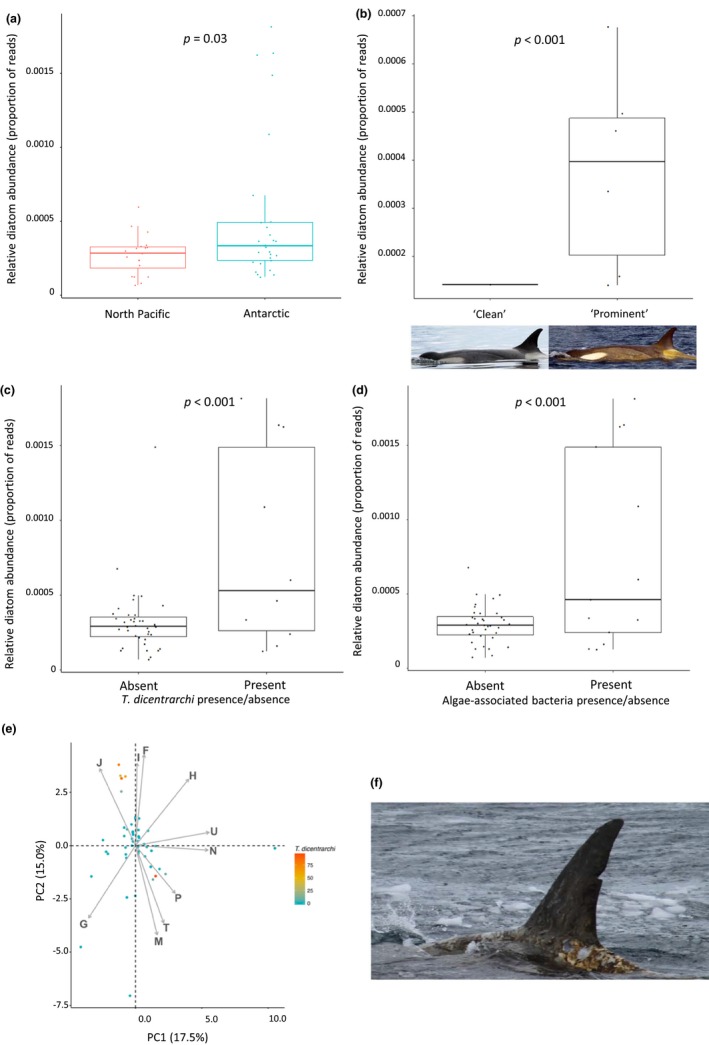
The influence of diatom abundance on skin microbiome community composition and microbial functional profiles. (a) Relative diatom abundance is significantly higher in Antarctic killer whales than North Pacific whales, but this is largely driven by a subset of outlier Antarctic individuals. (b) Within Antarctic *type B1* and *type B2* specimens, the relative diatom abundance is significantly associated with skin coloration of the host killer whale, with the yellowish hue being a reliable indicator of diatom load. Inset images are of the same *type B2* killer whale individual displaying extreme variability in diatom coverage, both photographs by John Durban. Relative diatom abundance is significantly associated with (c) the presence of *Tenacibaculum dicentrarchi* and (d) several algae‐associated bacteria, including *T. dicentrarchi*. (e) PCA of variation in functional COGs between individuals, coloured by *T. dicentrarchi* abundance. Individuals with high relative abundances of *T. dicentrarchi* generally cluster with high values in principal component 2. The top 10 COGs contributing to PCA variation are shown in grey arrows (J: translation, ribosomal structure and biogenesis, I: lipid transport and metabolism, F: nucleotide transport and metabolism, H: coenzyme transport and metabolism, U: intracellular trafficking, secretion and vesicular transport, N: cell motility, P: inorganic ion transport and metabolism, T: signal transduction mechanisms, M: cell wall membrane envelope biogenesis, G: carbohydrate transport and metabolism). (f) Photograph of a *type B1* killer whale in the Gerlache Strait of the Antarctic Peninsula on the 4 December 2015 with high diatom coverage and poor skin condition. Photograph by Conor Ryan

Both approaches to diatom identification produced concordant results (Supporting Information Figure S10, Table S5). Antarctic ecotypes had a significantly higher abundance of diatom DNA than North Pacific ecotypes (β = 0.65, *SE* = 0.29, *p* = 0.03; Figure 4a). Individuals with “prominent” yellow coloration showed higher diatom abundance (Figure 4b), supporting the link between skin colour and diatom presence in Antarctic killer whales. Furthermore, the abundance of diatom DNA per sample was significantly positively correlated with the abundance and presence of *T. dicentrarchi* reads (number of *T. dicentrarchi* reads: β = 0.014, *SE* = 0.003, *p* = <0.001; presence of *T. dicentrarchi*: β = 0.915, *SE* = 0.207, *p* < 0.001; Figure 4c) and the presence of at least one algae‐associated bacterial taxon (*β* = 0.98, *SE* = 0.17, *p* = <0.001; Figure 4d).

### 3.5 Functional analysis

In the read‐based functional analysis, a total of 3,611,441 reads mapped to eggNOG functions and 1,440,371 reads mapped to SEED functions. In the contig‐based functional analysis, we identified 56,042 potential genes in our metagenome, out of which eggnog function was assigned to 35,182. Both approaches identified energy production and conversion (class C) and amino acid metabolism and transport (class E) as the most abundant eggnog functions in our data set. The eggnog PCA revealed high variability between individuals (Figure 4e); however, a cluster of Antarctic whales was observed in principal component 2. These samples had high abundances of *T. dicentrarchi* (Figure 4e) and were associated with functions corresponding to the COG functional categories J (translation, ribosomal structure and biogenesis, β = 0.007, *SE* = 0.002, *p* = <0.001), F (nucleotide transport and metabolism, β = 0.005, *SE* = 0.002, *p* = 0.004) and I (lipid transport and metabolism, β = 0.004, *SE* = 0.002, *p* = 0.03). The same cluster of high abundance *T. dicentrarchi* Antarctic samples was also identified in the SEED PCA (Supporting Information Figure S11). These samples had increased numbers of reads mapping to DNA metabolism, amino acids and derivatives and cofactors/vitamins, although none of these functions was significantly correlated with *T. dicentrarchi* abundance.

## 4 DISCUSSION

Our study highlights that communities of exogenous or host‐associated microbiota can be genetically characterized from shotgun sequencing of DNA extracted from the host tissue. However, dedicated analysis and treatment of contamination are necessary and require careful consideration in studies such as this, whereby samples were not collected nor sequenced with the intention of genetically identifying microbiota. In such cases, the normal stringent control measures which are routine in microbial studies, such as the sequencing of blanks, may not be possible. We have therefore presented an array of approaches for estimating the proportion and sources of contamination and accounting for it in shotgun studies. Overall, our analyses suggest that with careful consideration, the mining of microbial DNA from host shotgun sequencing data can provide useful biological insights that inform future targeted investigations into microbiome composition and function under stringent laboratory conditions.

After carefully filtering our data, we were able to identify species interactions, ecological networks and community assembly of the microbes and diatoms that colonize killer whale skin by utilizing unmapped reads from shotgun sequencing data generated from skin biopsies. A key advantage of this approach over amplicon‐based sequencing is the ability to assess functional variation based on gene content and to identify taxa to species level (Koskella et al., 2017; Quince et al., 2017). However, despite ongoing efforts to describe bacterial species diversity, the breadth of the reference database is a limiting factor in the unbiased characterization of bacterial composition. Thus, taxa identified in our analyses are necessarily limited to species with available genomic information and in some cases are likely to represent their close phylogenetic relatives (Tessler et al., 2017). Hence, we refer to “taxa” rather than “species” where appropriate. We also demonstrate the impact of contamination on the low numbers of reads from true host‐associated microbes, which can dilute the signal of biologically meaningful variation among samples.

Social and geographical factors have been found to influence microbial diversity in terrestrial and semiterrestrial animals (Koskella et al., 2017). However, there is less understanding of how these factors interplay in a wide‐ranging social marine mammalian system (Nelson, Apprill, Mann, Rogers, & Brown, 2015). We found that beta diversity of the killer whale skin microbiome was significantly influenced by ecotype and latitude. Temperature has been shown to be a key determinant of marine microbial community structure at a global scale (Salazar & Sunagawa, 2017; Sunagawa et al., 2015). However, the effect of ecotype as the most important tested variable highlights the significance of social and phylogenetic factors in shaping microbiome richness and composition. In addition, it underscores that although killer whale skin is influenced by the local environment (Romano‐Bertrand, Licznar‐Fajardo, Parer, & Jumas‐Bilak, 2015), it represents a unique ecosystem that is separate from that of the surrounding habitat. Concordant with our results, a study of the microbiome of four captive killer whales and the sea water from their pool found that the skin microbiota were more diverse and phylogenetically distinct from the sea water microbial community (Chiarello et al., 2017). Killer whales are highly social mammals (Baird, 2000; Ford, 2009), and thus, they are likely to have a high potential for horizontal transfer of microbes between individuals during contact (Nelson et al., 2015). Ecotype‐specific social behaviour, organization and population structure, as well as other variables related to ecotype ecology, such as range size and diet (due to transmission of bacteria from different prey species; Wasimuddin et al., 2017), are all likely to affect the diversity of microbial species that individuals are exposed to and also influence the level of horizontal transfer of microbes between whales. The strong social philopatry in killer whales (Baird, 2000; Ford, 2009) and the phylogenetic and phylogeographical history of ecotypes is also likely to play a role, whereby due to limited social transmission between ecotypes, the phylogeny of bacterial species is likely to reflect that of the host (Ley, Lozupone, Hamady, Knight, & Gordon, 2008; but see Rothschild et al., 2018). It is also likely to be influenced by the host's evolutionary history, including secondary contact between ecotypes (Foote & Morin, 2016), where both vertical and horizontal transmissions of microbes between ecotypes are possible.

Despite the significance of “ecotype” as a driver of skin microbiome diversity in killer whales, at least 79% of the variation in the microbiome is unexplained by the factors considered in our models (Table 1). There is a strong overlap between ecotypes in the PCoA (Figure 3b), suggesting a shared core microbiome which may be partially shared with other cetacean species (Figure 2). Additionally, the PCoA shows substantial variation within ecotypes (Figure 3b), further highlighting the role of some other driver(s) of microbiome variation. Among Antarctic ecotypes, individual variation was associated with diatom presence and a discrete subnetwork of microbial taxa. The occurrence of a “yellow slime” attributed to diatoms on the skin of whales, including killer whales, was recorded as early as a century ago (Bennett, 1920; Pitman et al., 2018). The extent of diatom adhesion on Antarctic whales is thought to correlate with latitude and the time the whale has spent in cold waters (Hart, 1935; Konishi et al., 2008). The skin microbiome of humpback whales has been reported to change through the Antarctic foraging season (Bierlich et al., 2018), and our SourceTracker analysis found that humpback whales sampled during the late foraging season (i.e., individuals who had presumably spent longer in the Southern Ocean waters at the time of sampling) had more similarity to Southern Ocean microbial communities than those collected during the early foraging season. This raises the intriguing question as to whether the time spent in the frigid Antarctic waters could be a driver of variation in the skin microbiome and diatom load of Antarctic killer whales.

Satellite tracking of Antarctic killer whale movements documented rapid return migrations to subtropical latitudes, in which individuals travelled up to 9,400 km in 42 days (Durban & Pitman, 2012, 2013). Based on the strong directionality and velocity of travel during these migrations, Durban and Pitman (2012) hypothesized that they were not associated with breeding or feeding behaviour. Instead, they argued that these migrations could be driven by the need to leave the frigid Antarctic waters and temporarily move to warmer waters, to allow for physiological maintenance including the regeneration of the outer skin layer (Durban & Pitman, 2012). The identification of the same individuals in Antarctic waters, sometimes with a thick accumulation of diatoms, and at other times appearing “clean,” supports the hypothesis that skin regeneration is an intermittent rather than continuous process (Durban & Pitman, 2012).

We present genetic support for the hypothesis of Durban and Pitman (2012) that “clean” and yellow‐tinted *type B1* and *B2* killer whales represent differences in diatom load. In addition, we provide the first evidence that the extent of diatom coverage is also associated with significant variation in the skin microbiome community. We found that Antarctic killer whales with the highest diatom abundance also had skin microbiomes most similar to Southern Ocean microbial communities, suggesting that at the time of sampling, these individuals had spent longer in the Antarctic waters, consistent with the hypothesis that diatom coverage accumulates with time spent in the cold Southern Ocean waters. Perhaps most significantly, diatom abundance was positively correlated with the abundance of *T*. *dicentrarchi*, a known pathogen in several fish species, which is associated with skin lesions and severe tail and fin rot (Avendaño‐Herrera et al., 2016; Habib et al., 2014; Piñeiro‐Vidal, Gijón, Zarza, & Santos, 2012).

Our analyses revealed that samples with high abundances of *T*. *dicentrarchi* show distinct functional profiles. Functional analyses remain exploratory at this stage, constrained by the difficulty of translating broad functional categories into biological meaning. However, with more data that link individual health status and microbiome composition, functional analyses may provide a tool for identifying individuals at risk. Therefore, whether *T*. *dicentrarchi* represents a pathogen to killer whale hosts remains unknown. Type *B1* killer whales in apparently poor health and with heavy diatom loads have been observed with severe skin conditions (skin peeling and lesions; Figure 4f); however, *Tenacibaculum* sp. have been reported in up to 95% of humpback whales sampled in recent studies, which included apparently healthy individuals (Apprill, Mooney, Lyman, Stimpert, & Rappé, 2011, Apprill et al., 2014; Bierlich et al., 2018). Skin maintenance may thus represent a balancing act for Antarctic killer whales of managing the costs of pathogen load, thermal regulation, reduced foraging time and long‐range movement. Research into the skin microbiome should therefore continue to form a component of the ongoing holistic and multidisciplinary research programme to investigate the health of Antarctic killer whale populations and more broadly in studies on the health of marine mammals (e.g., Apprill et al., 2014; Raverty et al., 2017).

Ongoing field efforts provide the opportunity to further explore the relationships and interactions between killer whale hosts, their skin microbiome, other exogenous symbionts such as diatoms and the environment. Our community‐based analyses suggest the presence of a distinct environmental taxa network centred on *P. haloplanktis* as a driving taxon (Figure 3c). Collection and metagenomic characterization of environmental samples, such as sea water, alongside host biological samples would allow further explorations into the contribution of local ecological factors to the host microbiome. As a means of reducing the impact of contamination with DNA from laboratory environment, microbiome characterization can be conducted by means of RNA sequencing. This has an additional advantage of generating metatranscriptomic data, which, in combination with the metagenomic data, can facilitate the comparison/contrast between community function (using RNA transcript) and community taxonomic composition (using DNA sequence; Koskella et al., 2017). This may further reduce the potential impact of common laboratory contaminants, allowing the exploration of the bacterial functional repertoire that is in use in a given ecological context, including reconstruction of metabolic pathways (Bashiardes, Zilberman‐Schapira, & Elinav, 2016). Contamination in the laboratory could be further controlled for and characterized through inclusion of extraction, library preparation and PCR blanks as negative controls (Lusk, 2014; Salter et al., 2014) and measures such as double indexing (Kircher, Sawyer, & Meyer, 2011; Rohland & Reich, 2012; van der Valk, Vezzi, Ormestad, Dalén, & Guschanski, 2018), which can then inform the emerging downstream filtering methods for separating true microbiomes from contamination (Delmont & Eren, 2016; Davis et al., 2017). Lastly, the advances in long‐read sequencing using portable nanopore‐based platforms make it possible to generate data suitable for reconstructing complete bacterial genomes while in the field (Parker, Helmstetter, Devey, Wilkinson, & Papadopulos, 2017), including in the Antarctic (Johnson, Zaikova, Goerlitz, Bai, & Tighe, 2017). This is a promising development with respect to improving the breadth of host taxa from which bacterial taxa are derived and should improve future mapping of metagenomics data and taxonomic assignment.

## AUTHOR CONTRIBUTIONS

R.H., J.B., T.V. and A.A. analysed the data; J.W.D. and H.F. conducted the photographic grading. A.F. and K.G. conceived and coordinated the study, which was developed from a suggestion by Gerald Pao of the Salk Institute. J.W.D., H.F., R.W.B., M.B.H. and P.W. were involved in sample collection, and DNA was extracted by K.M.R. R.H., J.B., A.F. and K.G. wrote the manuscript with input from T.V., A.A., J.W.D., H.F., R.W.B., M.T.P.G., P.A.M. and J.B.W.W.

## Data Availability

All sequencing data are archived at the European Nucleotide Archive, www.ebi.ac.uk/ena, accession numbers: ERS554424–ERS554471. Scripts and metadata are available in Dryad repository https://doi.org/10.5061/dryad.c8v3rv6.
