# Supplementary material for: Host‐derived population genomics data provides insights into bacterial and diatom composition of the killer whale skin
Source: Mol Ecol. 2018 Oct 24;28(2):484–502. doi: 10.1111/mec.14860 (PMC6487819; doi:10.1111/mec.14860)
Supplement: Supplementary file 1 [file MEC-28-484-s001.docx]

**Supplemental Information for:**

**Host-derived population genomics data provides insights into bacterial and diatom composition of the killer whale skin**

Rebecca Hooper, Jaelle C. Brealey, Tom van der Valk, Antton Alberdi, John W. Durban, Holly Fearnbach, Kelly M. Robertson, Robin W. Baird, M. Bradley Hanson, Paul Wade, M. Thomas P. Gilbert, Phillip A. Morin, Jochen B.W. Wolf,

Andrew D. Foote, Katerina Guschanski

**Table of Contents:**

| **Figure S1** | Page 2 |
| --- | --- |
| **Figure S2** | Page 3 |
| **Figure S3** | Page 4 |
| **Figure S4** | Page 5 |
| **Figure S5** | Page 6 |
| **Figure S6** | Page 7 |
| **Figure S7** | Page 8 |
| **Figure S8** | Page 9 |
| **Figure S9** | Page 11 |
| **Figure S10** | Page 12 |
| **Figure S11** | Page 13 |
| **Table S1** | Page 14 |
| **Table S2** | Page 16 |
| **Table S3** | Page 18 |
| **Table S4** | Page 19 |
| **Table S5** | Page 20 |
| **Supplementary methods** | Page 21 |


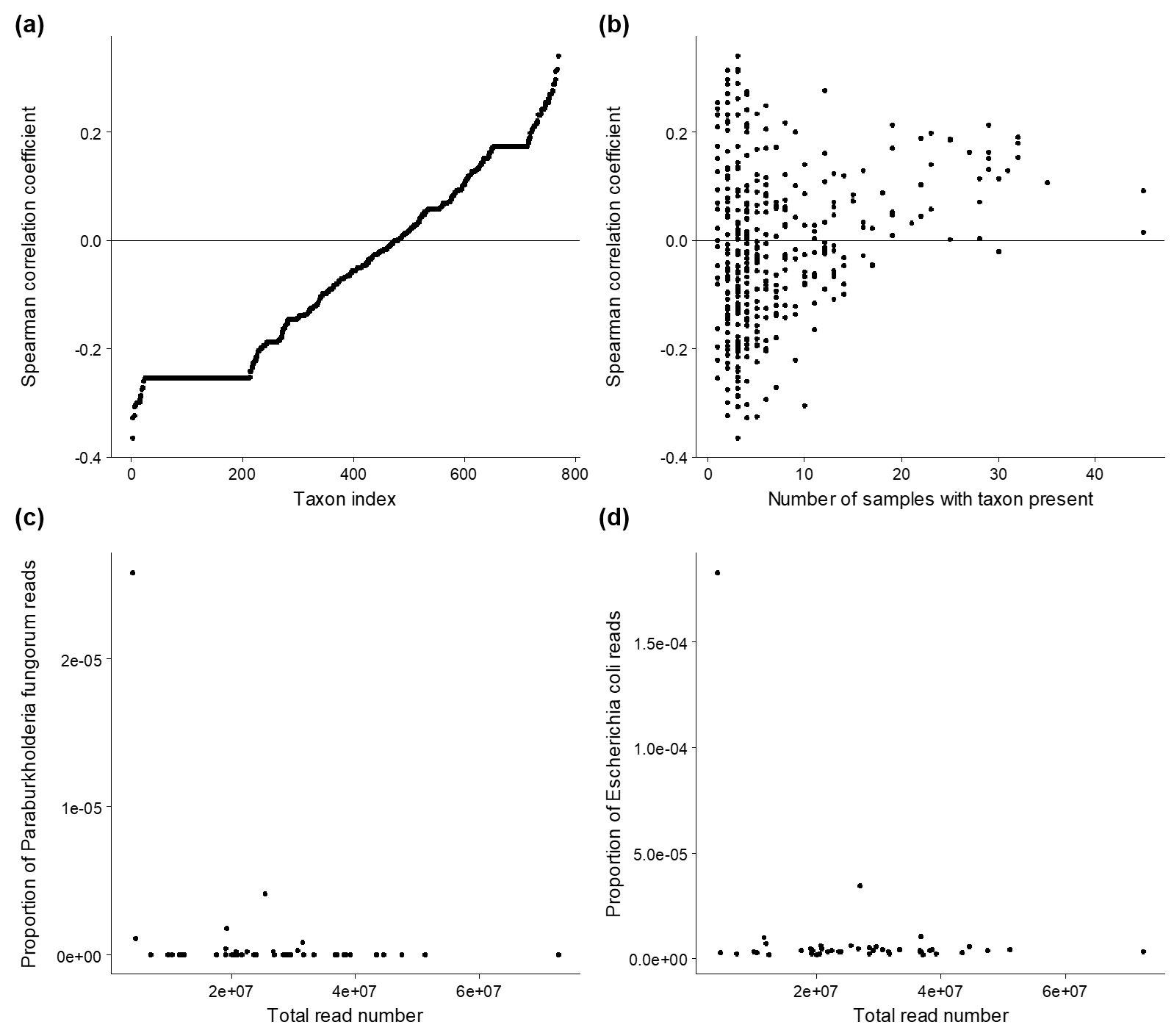


(e)

**Figure S1.** Correlations between proportion of reads per taxon and total read number (prior to removal of PCR duplicates)**.** Contaminating sequences included in the library PCR would be expected to represent a higher proportion of the total reads in libraries with low sample DNA quantity. (a) Spearman correlation coefficients were calculated for each taxon however, after adjustment for multiple comparisons, correlations were not significant for any species. (b) Negative correlations were not observed in the majority of taxa present in at least half the samples. (c) The species with the most negative correlations, including *Paraburkholderia fungorum* were largely driven by the lowest coverage sample, a type B1 individual (ID 124047). (d) One of the most frequently detected species, *Escherichia coli*, a common laboratory contaminant, had a correlation close to zero and followed a similar pattern, with low proportions overall and individual 124047 an outlier. (e) Distribution of correlation coefficients against P-values, line indicates significant results prior to adjustment for multiple comparisons.

**Figure S1.** **Correlations between proportion of reads per taxon and total read number** (prior to removal of PCR duplicates)**.** Contaminating sequences included in the library PCR are expected to represent a higher proportion of the total reads in libraries with low sample DNA quantity. (a) Spearman correlation coefficients for each taxon. After adjustment for multiple comparisons, correlations were not significant for any species. (b) Negative correlations were not observed in the majority of bacterial taxa present in at least half the samples. (c) The species with the strongest negative correlations, including *Paraburkholderia fungorum* were largely driven by the lowest coverage sample (ID B1_124047). (d) One of the most frequently detected species, *Escherichia coli*, a common laboratory contaminant, had a correlation close to zero, with low proportions overall. B1_124047 is an outlier. (e) Distribution of correlation coefficients against P-values, line indicates significant results prior to adjustment for multiple comparisons.

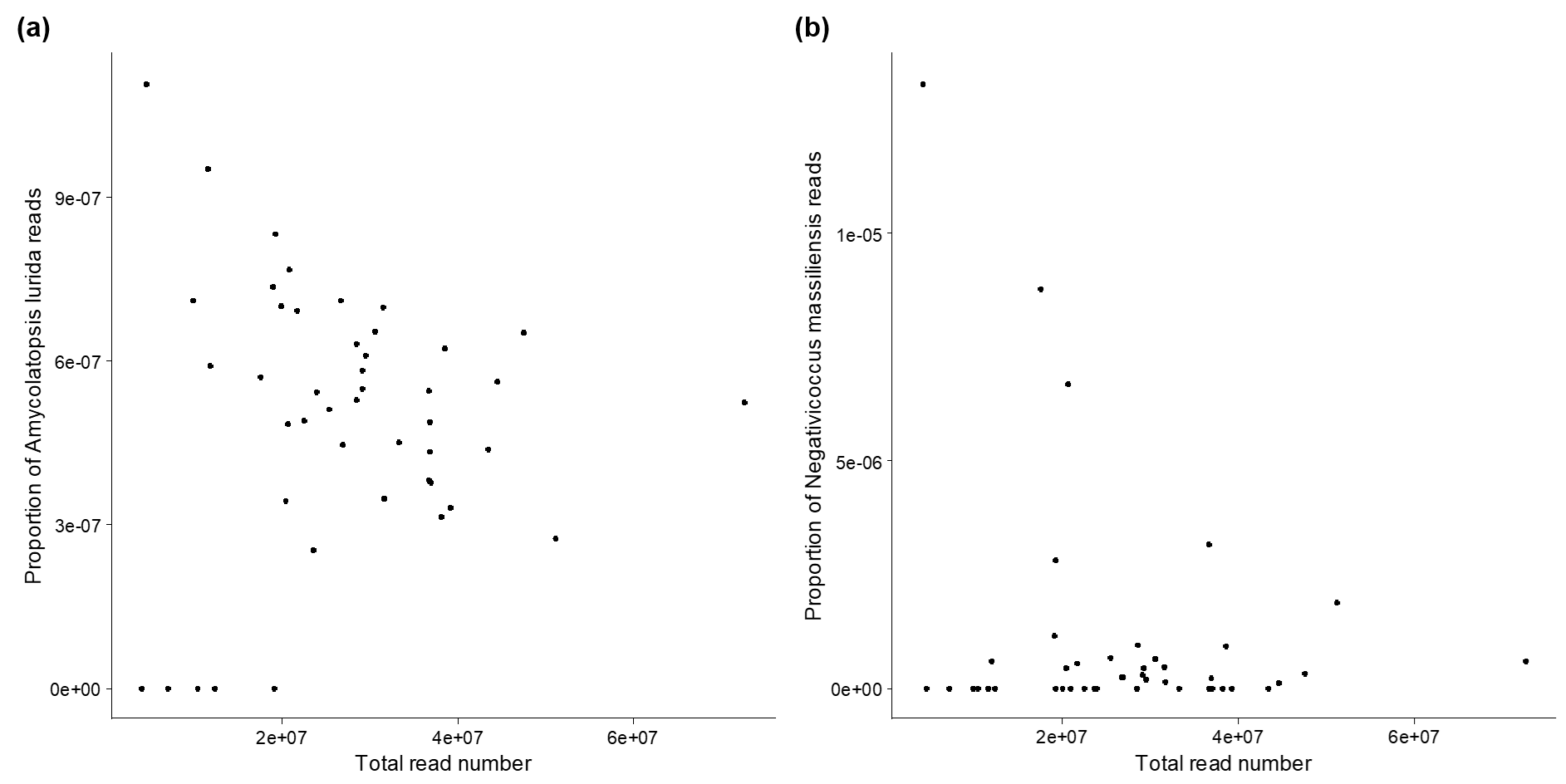


**Figure S2.** Taxa with a significant negative correlation between proportion of reads per taxon and total read number, prior to removal of PCR duplicates. The most common potential contaminants were filtered out during the removal of PCR duplicates step in our data processing pipeline. The MALT parameters used for the last common ancestor algorithm to assign taxonomy required a minimum of five uniquely mapping reads per taxon to designate that species as present in the data. Thus, potential contaminant taxa present in the dataset consisting predominantly of PCR duplicates did not meet this threshold. 149 taxa were identified only in the dataset containing PCR duplicates, including (a) *Amycolatopsis lurida* and (b) *Negativicoccus massiliensis*. Both tended to increase in relative abundance (proportion of reads mapping to the species over total read number in library) as total read number of the sample decreased, suggesting that they were contaminants sequenced to higher levels in samples with low coverage endogenous DNA.

**a**

**b
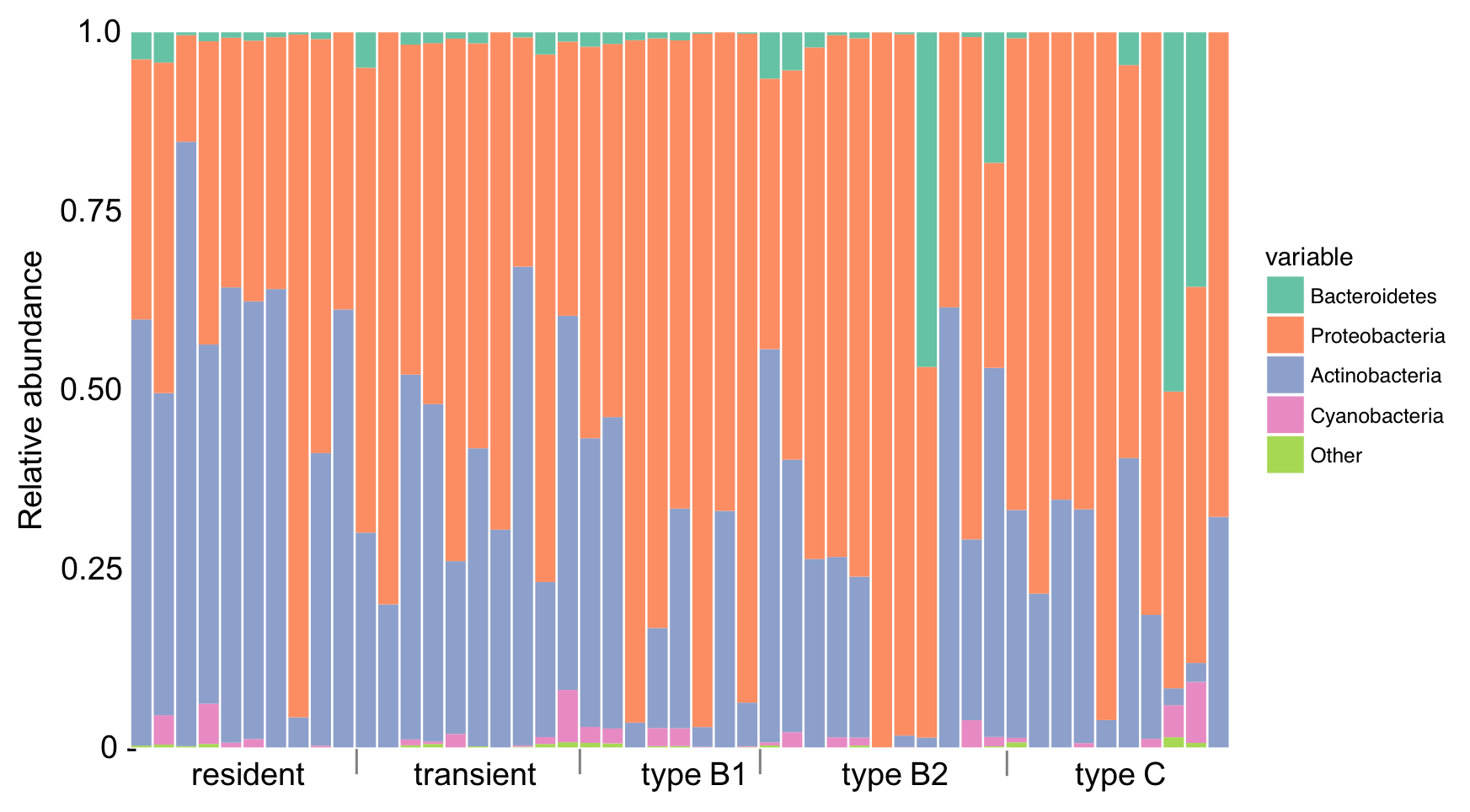
**


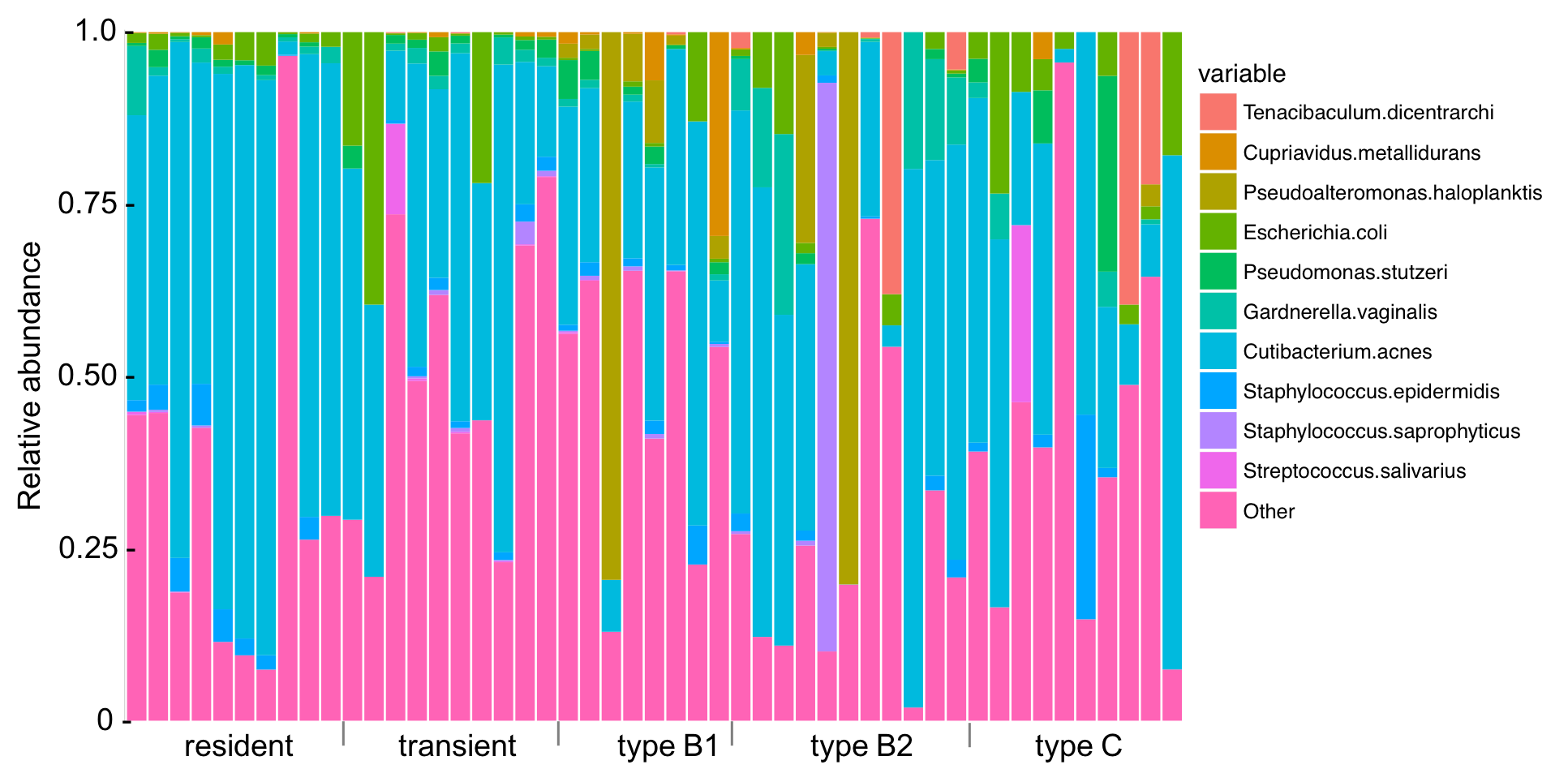
**Figure S3.** Relative abundances of bacterial taxa including *C*. *acnes* **(a)** at the phylum level, and **(b)** at the species level. Taxa representing >25% of the mapped bacterial reads in any one sample are indicated in the legends, all other are binned as ‘Other**’.** Note that Bacteroidetes (which include *T*. *dicentrarchi*), Proteobacteria and Actinobacteria, have all previously been identified as a component of whale species skin microbiomes (Appril et al., 2014; Shotts et al., 1990).

**
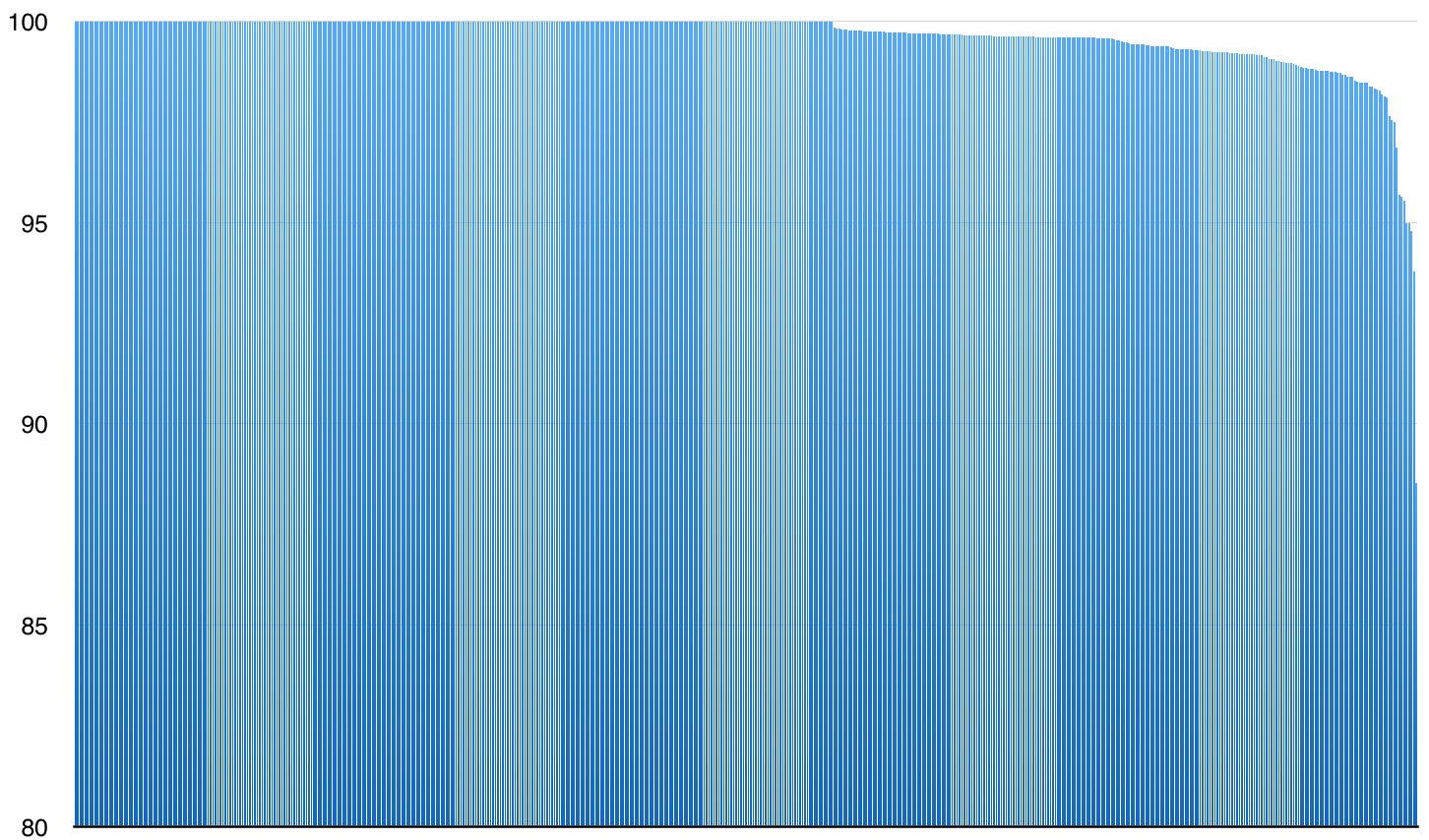
**

**Figure S4.** Percentage identity (*y*-axis) to the human-derived *C*. *acnes* reference genome of 527 contigs identified by MGMapper (each contig is represented by a column along the *x*-axis). Note the scale on the y-axis that starts at 80%.

**Figure S5.** Read counts for the ten most commonly identified bacterial taxa per lane for sample B1_124038. The proportions of sequencing reads assigned to each taxon were relatively consistent across sequencing lanes, see Coefficient of Variation (CV) values above the read count bars for each bacterial taxon. This consistency across sequencing lanes suggests that if *C*. *acnes* is a contaminant, then it was probably introduced during extraction or library building.


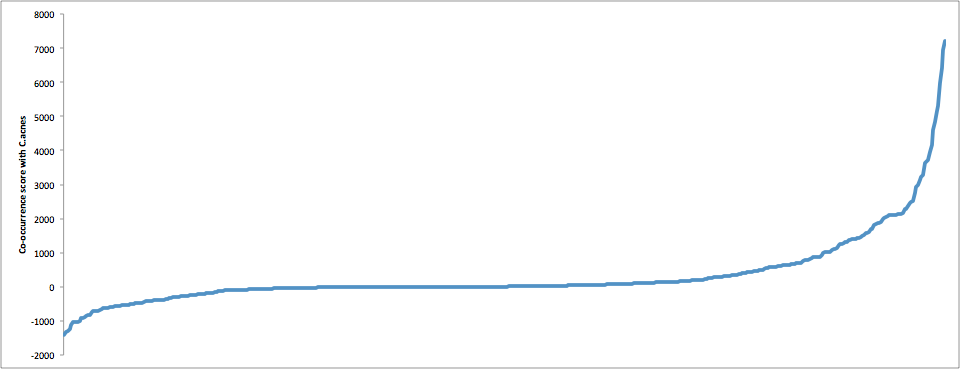


**Figure S6.**

Pairwise co-occurrence scores of *Cutibacterium acnes* and all other identified species. All species with a co-occurrence score above absolute 1000 were removed, corresponding to 10% of taxa, given their strong relationship with a likely contaminant.


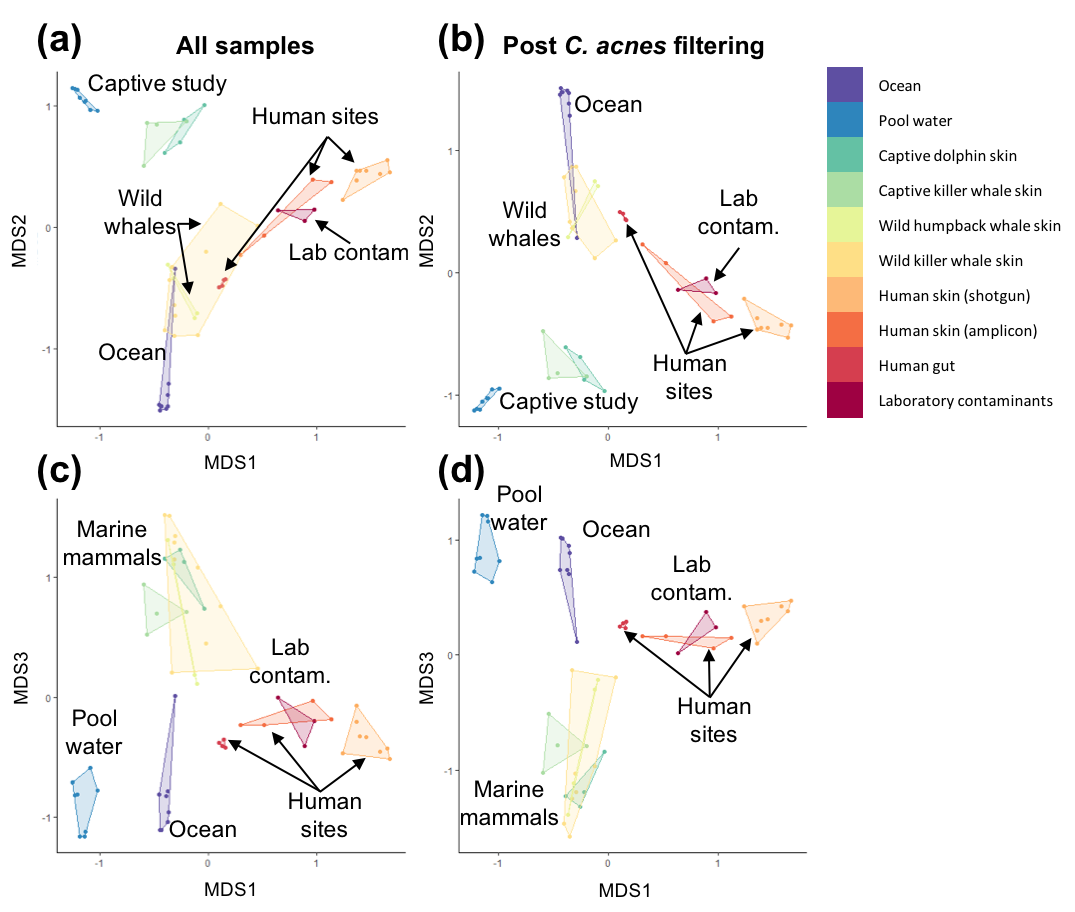


**Figure S7.** Principal coordinates analysis of Bray-Curtis relative abundance based distances for comparative microbiome datasets and wild killer whale samples with > 50 16S taxonomy assigned reads before (a,c) and after (b,d) filtering of *C. acnes* associated taxa from the wild killer whale data. (a-b) show the first versus second dimensions, (c-d) show the first versus third dimensions.


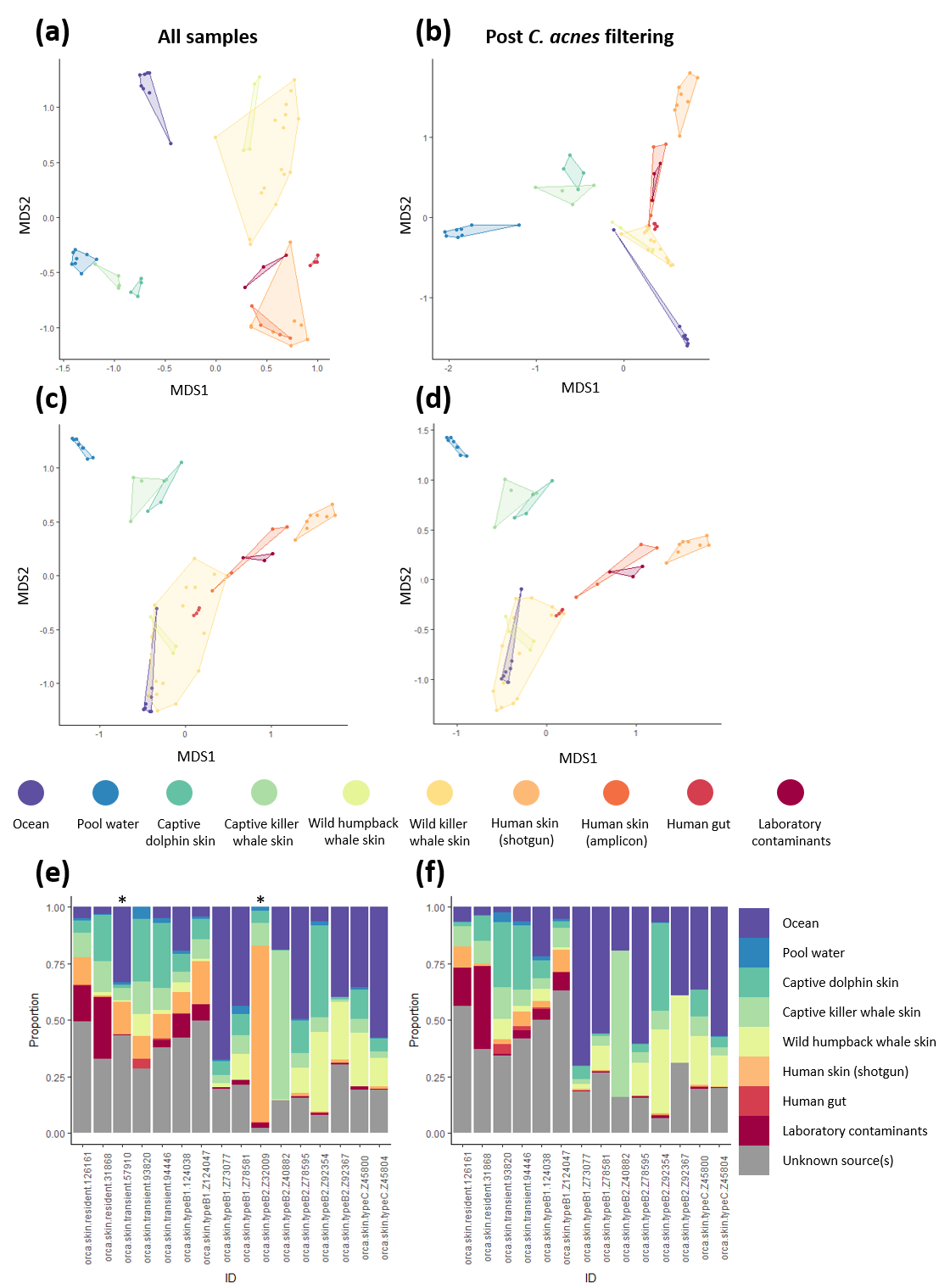


**Figure S8.** Composition of the wild killer whale skin microbiomes and other published microbiomes, for samples with ≥ 20 taxonomy assigned 16S reads. Principal coordinates analysis of Jaccard binary presence/absence distances (a-b) and Bray-Curtis relative abundance based distances (c-d) before (a,c) and after (b,d) filtering of *C. acnes* associated taxa from the wild killer whale data. Proportions of sources contributing to each killer whale sample from SourceTracker analysis before (e) and after (f) filtering of *C. acnes* associated taxa. * in (e) indicates samples that were excluded after *C. acnes* filtering due to low read numbers.


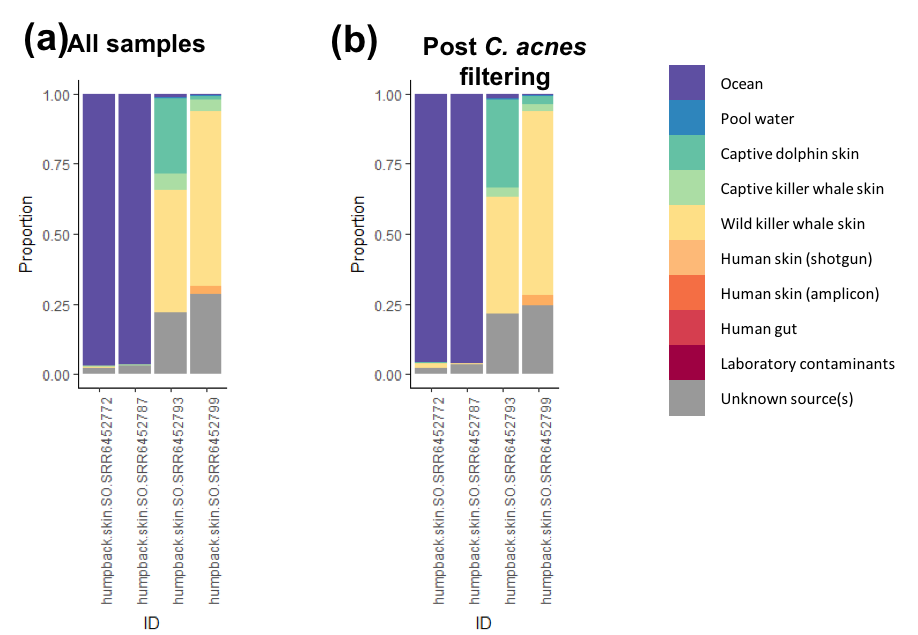


**Figure S9.** SourceTracker analyses of the four humpback whale skin microbiomes. Wild killer whales are included as sources before (a) and after (b) filtering *C. acnes* associated taxa, with ≥ 50 16S taxonomy assigned reads.


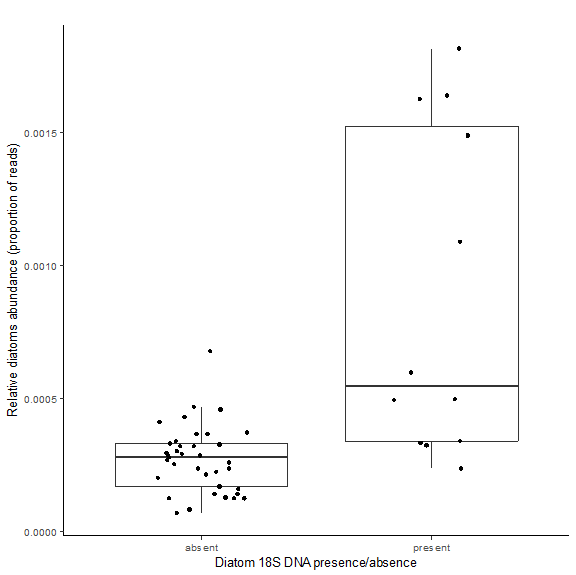


**Figure S10.** Comparison of diatom identification approaches. Individuals with diatoms 18S DNA (identified by reference-based OTU clustering to the Silva 18S reference database in QIIME) had higher relative abundances of diatoms reads (identified through MALT alignment to a custom diatoms reference database).


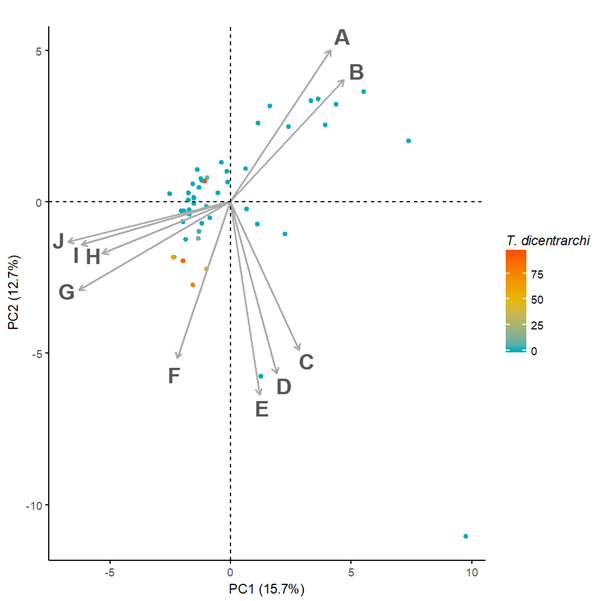


**Figure S11.** PCA of variation in functional SEED subsystems between individuals, coloured by *T. dicentrarchi* relative abundance. The top 10 subsystems contributing to PCA variation are shown in grey arrows (A: respiration, B: mitochondrial electron transport system in plants, C: RNA metabolism, D: transcriptional regulation, E: cell division and cell cycle, F: DNA metabolism, G: unclassified, H: amino acids and derivatives, I: cofactors, vitamins, prosthetic groups and pigments, J: miscellaneous).

**Table S1. Estimates of human contamination.** Percentage of filtered reads mapping to the human genome

| **Sample** | **Total reads (after filtering)** | **Reads mapping to killer whale** | **Reads mapping to human** | **Human contamination (%)** |
| --- | --- | --- | --- | --- |
| resident_126158 | 71296920 | 67476122 | 38172 | 0.054 |
| resident_126161 | 51717046 | 49820228 | 37591 | 0.073 |
| resident_126163 | 79657106 | 76587775 | 42109 | 0.053 |
| resident_126165 | 23449198 | 22271866 | 24411 | 0.104 |
| resident_126167 | 70046000 | 67449705 | 8156 | 0.012 |
| resident_126169 | 65131956 | 62689028 | 7491 | 0.012 |
| resident_126178 | 69707699 | 67242117 | 8899 | 0.013 |
| resident_31868 | 54739155 | 52597619 | 11760 | 0.021 |
| resident_35322 | 58372613 | 56191943 | 10381 | 0.018 |
| resident_62250 | 29741836 | 28406408 | 5107 | 0.017 |
| transient_28549 | 9098208 | 8327162 | 2113 | 0.023 |
| transient_40246 | 17689409 | 17017866 | 2022 | 0.011 |
| transient_57910 | 56602921 | 54448006 | 336625 | 0.595 |
| transient_57919 | 49213098 | 47496738 | 22742 | 0.046 |
| transient_62471 | 38801446 | 36729207 | 10427 | 0.027 |
| transient_67975 | 42565709 | 41129490 | 16051 | 0.038 |
| transient_79751 | 53716078 | 51777082 | 5782 | 0.011 |
| transient_79759 | 93599028 | 88934956 | 41805 | 0.045 |
| transient_93820 | 35393880 | 33488956 | 33665 | 0.095 |
| transient_94446 | 103920966 | 99845173 | 191026 | 0.184 |
| typeB1_124038 | 54359944 | 51377650 | 45705 | 0.084 |
| typeB1_124047 | 5200537 | 4901285 | 282599 | 5.434 |
| typeB1_32005 | 26773108 | 25833873 | 2518 | 0.009 |
| typeB1_73077 | 72666047 | 48711839 | 19515 | 0.027 |
| typeB1_78580 | 38222599 | 35423771 | 13994 | 0.037 |
| typeB1_78581 | 18263842 | 17555517 | 21933 | 0.120 |
| typeB1_88344 | 42676405 | 41078880 | 4894 | 0.011 |
| typeB1_88347 | 74514685 | 52645243 | 176500 | 0.237 |
| typeB2_124036 | 34061926 | 31978162 | 5611 | 0.016 |
| typeB2_124043 | 38292054 | 36828793 | 5162 | 0.013 |
| typeB2_31884 | 82677056 | 79530541 | 7874 | 0.010 |
| typeB2_32009 | 87349658 | 84034490 | 17904 | 0.020 |
| typeB2_40882 | 62379635 | 60103826 | 8478 | 0.014 |
| typeB2_78595 | 65647529 | 62334767 | 15414 | 0.023 |
| typeB2_92354 | 24000642 | 22721625 | 3504 | 0.015 |
| typeB2_92362 | 5604139 | 5311113 | 1295 | 0.023 |
| typeB2_92363 | 50350550 | 47602948 | 6875 | 0.014 |
| typeB2_92365 | 52256148 | 49667352 | 8617 | 0.016 |
| typeB2_92367 | 26604414 | 24119389 | 13336 | 0.050 |
| typeC_26614 | 58823018 | 56500854 | 6098 | 0.010 |
| typeC_26617 | 39909702 | 38187701 | 3102 | 0.008 |
| typeC_26619 | 77862637 | 74762206 | 10912 | 0.014 |
| typeC_26620 | 57380615 | 55101572 | 6080 | 0.011 |
| typeC_26623 | 65950746 | 63352072 | 5295 | 0.008 |
| typeC_26626 | 18489779 | 17764629 | 1879 | 0.010 |
| typeC_26627 | 52411016 | 50350398 | 6180 | 0.012 |
| typeC_45800 | 42999673 | 41089861 | 4410 | 0.010 |
| typeC_45804 | 60648518 | 57758731 | 5829 | 0.010 |
| typeC_88353 | 36794227 | 35268338 | 3740 | 0.010 |

**Table S2.** Datasets used for comparison of microbiomes in SourceTracker and principal coordinates analyses.

| **Environment** | **SRA/ENA** | **Project accession** | **Sequencing strategy (locus)** | **Reference** | **SampleID** | **# 16S read** | **# reads assigned taxonomy** | **Used in Source-Tracker** |
| --- | --- | --- | --- | --- | --- | --- | --- | --- |
| Human gut (Stool) | SRA | PRJNA 48479 | Shotgun | Llody-Price et al. 2017 | SRR1564226 | 2154 | 1942 | Yes |
|  |  |  |  |  | SRR1564316 | 2204 | 1853 |  |
|  |  |  |  |  | SRR1564319 | 1743 | 1519 |  |
|  |  |  |  |  | SRR1565914 | 1758 | 1466 |  |
| Human skin (Cheek, sebaceous) | SRA | PRJNA46333 | Shotgun | Oh et al. 2014 | MET0061 | 382 | 228 | Yes |
|  |  |  |  |  | MET0184 | 1390 | 714 |  |
|  |  |  |  |  | MET0256 | 1294 | 858 |  |
|  |  |  |  |  | MET0307 | 657 | 470 |  |
| Human skin (Forearm, dry) |  |  |  |  | MET0243 | 7296 | 3488 | No |
|  |  |  |  |  | MET0269 | 4823 | 1982 |  |
|  |  |  |  |  | MET0286 | 4056 | 2328 |  |
|  |  |  |  |  | MET0320 | 3448 | 1216 |  |
| Human skin (Forehead, sebaceous) | SRA | PRJNA295605 | Amplicon (16S V4) | Meisel et al. 2016 | SRR5219908 | 6675 | 5611 | No |
|  |  |  |  |  | SRR5219909 | 85151 | 82906 |  |
|  |  |  |  |  | SRR5219911 | 5081 | 4181 |  |
|  |  |  |  |  | SRR5219915 | 38589 | 36992 |  |
| Laboratory contaminants (Sterile water) | ENA | PRJEB 7055 | Amplicon (16S V1V2) | Salter et al. 2014 | ERR584320 | 420 | 353 | Yes |
|  |  |  |  |  | ERR584333 | 13359 | 10360 |  |
|  |  |  |  |  | ERR584341 | 19 | 18 |  |
|  |  |  |  |  | ERR584348 | 8061 | 6433 |  |
| Ocean water (Southern Ocean) | ENA | PRJEB 1787 | Shotgun | Sunagawa et al. 2015 | ERS491095 | 61539 | 26451 | Yes |
|  |  |  |  |  | ERS491107 | 1942 | 1461 |  |
|  |  |  |  |  | ERS491001 | 48246 | 25655 |  |
|  |  |  |  |  | ERS491044 | 53947 | 30224 |  |
| Ocean water (North Pacific Ocean) |  |  |  |  | ERS493340 | 37657 | 24167 | No |
|  |  |  |  |  | ERS493431 | 28346 | 19185 |  |
|  |  |  |  |  | ERS493300 | 36134 | 23022 |  |
|  |  |  |  |  | ERS493390 | 49479 | 34306 |  |
| Captive dophin pool water | SRA | PRJNA391940 | Amplicon (16S V3V4) | Chiarello et al. 2017 | SRR5757522 | 5135 | 631 | No |
|  |  |  |  |  | SRR5757549 | 10152 | 2384 |  |
|  |  |  |  |  | SRR5757552 | 9312 | 1104 |  |
|  |  |  |  |  | SRR5757553 | 13401 | 1556 |  |
| Captive killer whale pool water |  |  |  |  | SRR5757534 | 14583 | 3491 | Yes |
|  |  |  |  |  | SRR5757535 | 13581 | 1888 |  |
|  |  |  |  |  | SRR5757537 | 13997 | 1570 |  |
|  |  |  |  |  | SRR5757548 | 15319 | 3672 |  |
| Captive dolphin skin (Dorsal fin) |  |  |  |  | SRR5757541 | 11884 | 3136 | Yes |
|  |  |  |  |  | SRR5757545 | 14860 | 3994 |  |
|  |  |  |  |  | SRR5757547 | 6323 | 1482 |  |
|  |  |  |  |  | SRR5757559 | 4918 | 997 |  |
| Captive killer whale skin (Dorsal fin) |  |  |  |  | SRR5757523 | 7117 | 1925 | Yes |
|  |  |  |  |  | SRR5757525 | 9702 | 3572 |  |
|  |  |  |  |  | SRR5757529 | 36852 | 2737 |  |
|  |  |  |  |  | SRR5757563 | 17892 | 8240 |  |
| Wild humpback whale skin | SRA | PRJNA395930 | Amplicon (16S V4) | Bierlich et al. 2018 | SRR6452772 | 22032 | 20638 | Yes |
|  |  |  |  |  | SRR6452787 | 24125 | 23220 |  |
|  |  |  |  |  | SRR6452793 | 18689 | 1428 |  |
|  |  |  |  |  | SRR6452799 | 10289 | 1451 |  |
| Wild killer whale skin | NA | NA | Shotgun | This study | resident_126161 | 46 | 25 | Used as 'sink' samples |
|  |  |  |  |  | resident_126165 | 30 | 18 |  |
|  |  |  |  |  | resident_31868 | 68 | 50 |  |
|  |  |  |  |  | transient_57910 | 47 | 22 |  |
|  |  |  |  |  | transient_93820 | 37 | 23 |  |
|  |  |  |  |  | transient_94446 | 155 | 100 |  |
|  |  |  |  |  | typeB1_124038 | 80 | 34 |  |
|  |  |  |  |  | typeB1_Z124047 | 159 | 98 |  |
|  |  |  |  |  | typeB1_Z73077 | 273 | 165 |  |
|  |  |  |  |  | typeB1_Z78581 | 63 | 39 |  |
|  |  |  |  |  | typeB2_92365 | 37 | 14 |  |
|  |  |  |  |  | typeB2_Z32009 | 41 | 23 |  |
|  |  |  |  |  | typeB2_Z40882 | 3004 | 2894 |  |
|  |  |  |  |  | typeB2_Z78595 | 421 | 279 |  |
|  |  |  |  |  | typeB2_Z92354 | 124 | 89 |  |
|  |  |  |  |  | typeB2_Z92367 | 84 | 50 |  |
|  |  |  |  |  | typeC_Z45800 | 93 | 74 |  |
|  |  |  |  |  | typeC_Z45804 | 333 | 220 |  |
|  |  |  |  |  | resident_126158 | 10 | 4 | Excluded from ‘sink’ samples due to low read counts |
|  |  |  |  |  | resident_126163 | 8 | 2 |  |
|  |  |  |  |  | resident_126167 | 12 | 5 |  |
|  |  |  |  |  | resident_126169 | 5 | 2 |  |
|  |  |  |  |  | resident_126178 | 9 | 1 |  |
|  |  |  |  |  | resident_35322 | 12 | 4 |  |
|  |  |  |  |  | resident_62250 | 2 | 1 |  |
|  |  |  |  |  | transient_57919 | 8 | 3 |  |
|  |  |  |  |  | transient_62471 | 9 | 5 |  |
|  |  |  |  |  | transient_67975 | 16 | 9 |  |
|  |  |  |  |  | transient_79751 | 3 | 1 |  |
|  |  |  |  |  | transient_79759 | 18 | 6 |  |
|  |  |  |  |  | transient_28549 | 3 | 0 |  |
|  |  |  |  |  | transient_40246 | 1 | 0 |  |
|  |  |  |  |  | typeB1_Z32005 | 2 | 1 |  |
|  |  |  |  |  | typeB1_Z78580 | 14 | 2 |  |
|  |  |  |  |  | typeB1_Z88344 | 2 | 2 |  |
|  |  |  |  |  | typeB1_Z88347 | 15 | 7 |  |
|  |  |  |  |  | typeB2_Z124036 | 9 | 1 |  |
|  |  |  |  |  | typeB2_Z124043 | 2 | 0 |  |
|  |  |  |  |  | typeB2_Z31884 | 6 | 3 |  |
|  |  |  |  |  | typeB2_Z92362 | 0 | 0 |  |
|  |  |  |  |  | typeB2_Z92363 | 6 | 1 |  |
|  |  |  |  |  | typeC_26619 | 9 | 0 |  |
|  |  |  |  |  | typeC_Z26614 | 5 | 0 |  |
|  |  |  |  |  | typeC_Z26617 | 2 | 2 |  |
|  |  |  |  |  | typeC_Z26620 | 5 | 1 |  |
|  |  |  |  |  | typeC_Z26623 | 5 | 4 |  |
|  |  |  |  |  | typeC_Z26626 | 2 | 0 |  |
|  |  |  |  |  | typeC_Z26627 | 6 | 2 |  |
|  |  |  |  |  | typeC_Z88353 | 2 | 0 |  |

**Table S3.** The effect of subsampling data to 10x, 5x and 2x coverage on read counts for microbial taxa from the shotgun sequencing data of a 20x coverage Alaskan *resident* killer whale (Moura et al., 2016). Taxa found in at least one killer whale in our study are flagged in the rightmost column.


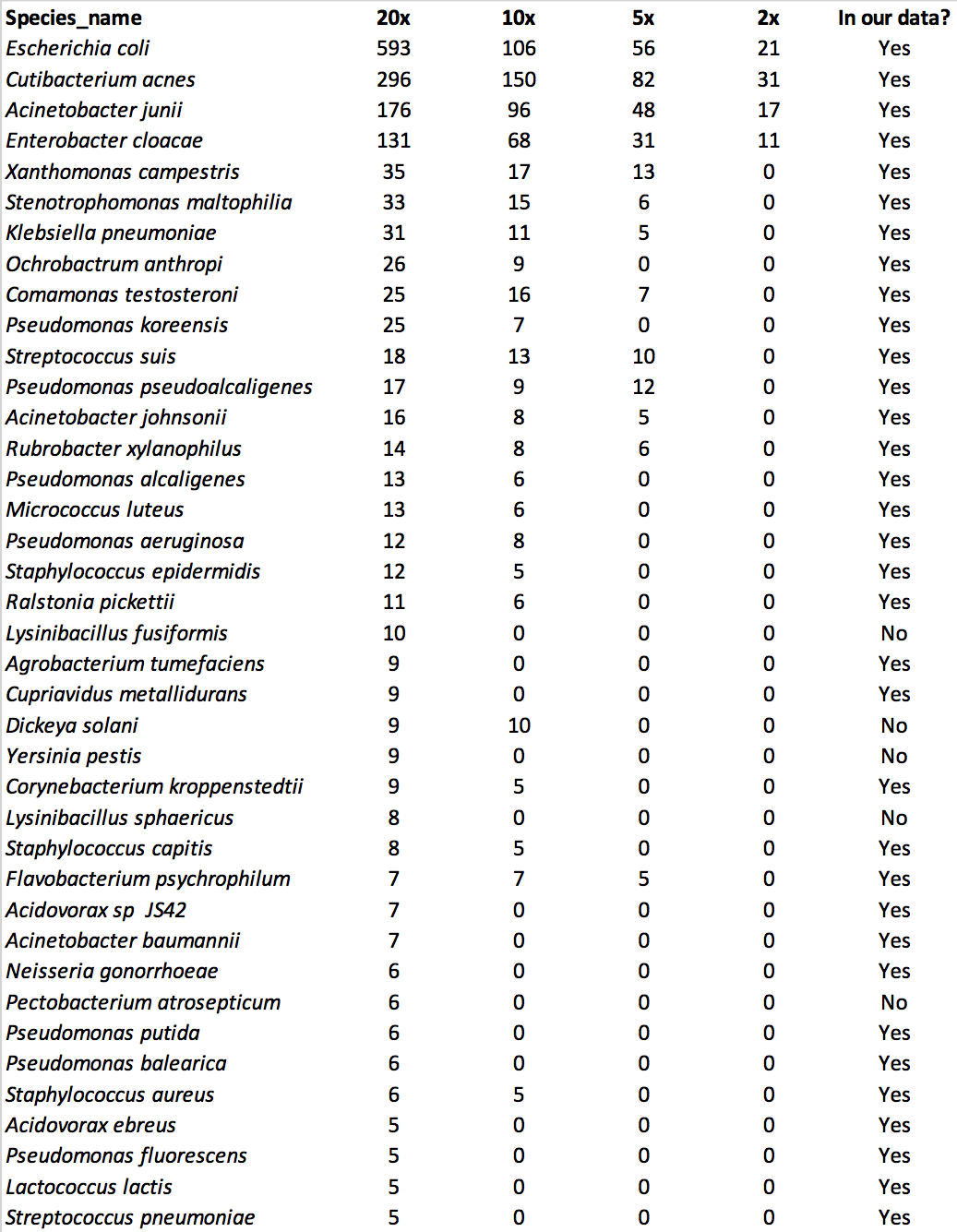


**Table S4.** The top four drivers on PCoA1 and PCoA2 of Figure 3.

| Species | PCoA1 |
| --- | --- |
| *Tenacibaculum dicentrarchi* | 0.896 |
| *Paraburkholderia fungorum* | 0.672 |
| *Kocuria palustris* | -0.890 |
| *Pseudomonas stutzeri* | -0.534 |
|  | PCoA2 |
| *Pseudoalteromonas haloplanktis* | 0.575 |
| *Pseudoalteromonas translucida* | 0.230 |
|  |  |
|  |  |

**Table S5.** Detection of diatom 18S DNA identified with the SILVA reference database.

|  | Diatom 18S DNA | | Estimate, standard error  and p value^1^ |
| --- | --- | --- | --- |
|  | **Present** | **Absent** |  |
| All individuals | 12 (24.5%) | 37 (75.5%) |  |
| Location |  |  | β = 1.20, SE = 0.81, p = 0.14 |
| North Pacific (n=20) | 3 (15.0%) | 17 (85.0%) |  |
| Antarctic (n=29) | 9 (31.0%) | 20 (69.0%) |  |
| Skin colouration |  |  | β = -190, SE = NA, p = 1 |
| Prominent yellow (n=6) | 4 (66.7%) | 2 (33.3%) |  |
| Clean (n=1) | 0 (0%) | 1 (100%) |  |
| Algae-associated bacteria |  |  | β = 2.81, SE = 0.86, p = 0.001 |
| Present (n=13) | 8 (61.5%) | 5 (38.5%) |  |
| Absent (n=36) | 4 (11.1%) | 32 (88.9%) |  |
| *Tenacibaculum dicentrarchi* |  |  | β = 2.34, SE = 0.88, p = 0.008 |
| Present (n=10) | 6 (60.0%) | 4 (40.0%) |  |
| Absent (n=39) | 6 (15.4%) | 33 (84.6%) |  |

^1^Binomial GLM adjusted for latitude, longitude, library complexity and human contamination levels.

SUPPLEMENTARY TEXT

**Read processing for assessing human-derived microbial contamination.**

Trimmomatic was used to remove Illumina sequencing adapters, all leading and trailing bases below quality score 15 in a sliding window of 4 base pairs, cutting once the average quality within the window fell below 15 [1]. After trimming, we removed all reads shorter than 30 base pairs. Since reads from repetitive regions have a higher change of false mappings, resulting in false identification of (human) contamination, we removed all reads with low to medium complexity by filtering out reads below entropy score of 50 using Prinseq [2]. We then mapped the filtered dataset against an index consisting of the human (GRCh38.p12), the phiX (NC_001422.1), the C. acnes (NC_006085.1) and the killer whale (Oorca1.1) reference genomes. The mapping against multiple references simultaneously allows for the identification of the best hit at the correct genome.

**Read processing for SourceTracker analyses.**

We downloaded four samples from each source from the SRA (<ftp://ftp-trace.ncbi.nih.gov/sra/> sra-instant/reads/ByRun/sra/SRR) or ENA (ftp://ftp.sra.ebi.ac.uk/vol1/fastq/) (see Table S2 for accessions). SRA files were converted to fastq files using the SRA Toolkit V2.8.2. Adapter-trimming and read length filtering (< 35bp) was conducted with AdapterRemoval V2.2.2 (Schubert, Lindgreen, & Orlando, 2016) and paired-end reads were collapsed during this step, where necessary. Duplicate sequences were merged with the in-house python scripts for shotgun studies, but not for amplicon-based studies, since removing PCR duplicates would result in the loss of all abundance information. Reads mapping to the PhiX reference genome were identified and removed as above. We filtered out reads mapping to the human reference genome GRCh38.p7 with BWA-mem and SAMtools in both published data and our samples. To be able to include sources with both 16S and shotgun data, we extracted reads mapping to the SILVA rRNA database release 128 (Quast et al. 2013) for all sources and for our killer whale data using BWA-mem V0.7.17 and SAMtools V1.8, retaining reads mapping at a quality score of 10. Closed-reference OTU picking was conducted with uclust (Edgar 2010) in QIIME V1.9.1 (Caporaso et al. 2010) against the SILVA 16S rRNA database, enabling reverse strand matching and with an OTU similarity of 97%. Genus-level taxonomy was assigned based on the SILVA 16S rRNA taxonomy (Yilmaz et al. 2014). Due to the low sequencing depth of our samples, only few reads mapped to the 16S locus (see Table S2 for 16S read counts). Therefore, we performed our analysis on subsets of samples with at least 50 reads for which taxonomy could be assigned. To provide an additional test, we relaxed the selection criterion and performed the same analyses also for samples with at least 20 taxonomy-assigned reads.

1. Bolger AM, Lohse M, Usadel B. 2014 Trimmomatic: a flexible trimmer for Illumina sequence data. *Bioinformatics* **30**, 2114–20. (doi:10.1093/bioinformatics/btu170)
2. Schmieder R, Edwards R. 2011 Quality control and preprocessing of metagenomic datasets. *Bioinformatics* **27**, 863–864. (doi:10.1093/bioinformatics/btr026)
